# Supplementary material for: System Approaches to Water, Sanitation, and Hygiene: A Systematic Literature Review
Source: Int J Environ Res Public Health. 2020 Jan 21;17(3):702. doi: 10.3390/ijerph17030702 (PMC7037755; doi:10.3390/ijerph17030702)
Supplement: Supplementary file 1 [file ijerph-17-00702-s001.pdf]

Article

Systems Approaches to Water, Sanitation and Hygiene: A Systematic Literature Review

Nicholas Valcourt[1,3,*], Amy Javernick-Will[1,3], Jeffrey Walters[2,3], Karl Linden[1,3]

[1] Department of Civil, Environmental, and Architectural Engineering, University of Colorado Boulder, Boulder, CO 80309
[2] College of Engineering, George Fox University, Newberg, OR 97132
[3] USAID Sustainable WASH Systems Learning Partnership
* Correspondence: Nicholas.Valcourt@Colorado.edu;

Received: date; Accepted: date; Published: date

Abstract: Endemic issues of sustainability in the Water, Sanitation and Hygiene (WASH) sector have led to the rapid expansion of 'system approaches' to for assessing the multitude of interconnected factors that affect WASH outcomes. However, the sector lacks a systematic analysis and characterization of the knowledge base for systems approaches, in particular how and where they are being implemented and what outcomes have resulted from their application. To address this need, we conducted a wide-ranging systematic literature review of systems approaches for WASH across the peer-reviewed, grey, and organizational literature. Our results show a myriad of methods, scope, and applications within the sector, but an inadequate level of information in the literature to evaluate the utility and efficacy of systems approaches for improving WASH service sustainability. Based on this analysis, we propose four recommendations for improving the evidence base including; diversifying methods that explicitly evaluate interconnections between factors within WASH systems, expanding geopolitical applications, improving reporting on resources required to implement given approaches, and enhancing documentation of effects of system approaches on WASH services. Overall, these findings provide a robust survey of the existing landscape of systems approaches for WASH and propose a path for future research in this emerging field.

Keywords: WASH; systems approaches; systematic literature review; grey literature

---

Supplementary Information

SI 1: PRISMA CHECKLIST

Table S1-1: Preferred Reporting Items for Systematic Reviews and Meta-Analyses (PRISMA) Checklist

SECTION/TOPIC	#	CHECKLIST ITEM	REPORTED ON PAGE #
**TITLE**			
**Title**	1	Identify the report as a systematic review, meta-analysis, or both.	Title
**ABSTRACT**			
**Structured summary**	2	Provide a structured summary including, as applicable: background; objectives; data sources; study eligibility criteria, participants, and interventions; study appraisal and synthesis methods; results; limitations; conclusions and implications of key findings; systematic review registration number.	Abstract
**INTRODUCTION**			
**Rationale**	3	Describe the rationale for the review in the context of what is already known.	Introduction
**Objectives**	4	Provide an explicit statement of questions being addressed with reference to participants, interventions, comparisons, outcomes, and study design (PICOS).	1.1 Study objectives
**METHODS**			
**Protocol and registration**	5	Indicate if a review protocol exists, if and where it can be accessed (e.g., Web address), and, if available, provide registration information including registration number.	2. Methods
**Eligibility criteria**	6	Specify study characteristics (e.g., PICOS, length of follow-up) and report characteristics (e.g., years considered, language, publication status) used as criteria for eligibility, giving rationale.	2.1 Literature ID
**Information sources**	7	Describe all information sources (e.g., databases with dates of coverage, contact with study authors to identify additional studies) in the search and date last searched.	2.1 Literature ID
**Search**	8	Present full electronic search strategy for at least one database, including any limits used, such that it could be repeated.	2.1 Table 1, search string formula
**Study selection**	9	State the process for selecting studies (i.e., screening, eligibility, included in systematic review, and, if applicable, included in the meta-analysis).	2.2 Selection of articles
**Data collection process**	10	Describe method of data extraction from reports (e.g., piloted forms, independently, in duplicate) and any processes for obtaining and confirming data from investigators.	2.2 Data extraction
**Data items**	11	List and define all variables for which data were sought (e.g., PICOS, funding sources) and any assumptions and simplifications made.	2.2.1; 2.2.2 Screening criteria
**Risk of bias in individual studies**	12	Describe methods used for assessing risk of bias of individual studies (including specification of whether this was done at the study or outcome level), and how this information is to be used in any data synthesis.	2.2.2.3 Application

**Summary measures**	13	State the principal summary measures (e.g., risk ratio, difference in means).	N/A
**Synthesis of results**	14	Describe the methods of handling data and combining results of studies, if done, including measures of consistency (e.g., I^2) for each meta-analysis.	N/A
**Risk of bias across studies**	15	Specify any assessment of risk of bias that may affect the cumulative evidence (e.g., publication bias, selective reporting within studies).	Conclusions / Limitations
**Additional analyses**	16	Describe methods of additional analyses (e.g., sensitivity or subgroup analyses, meta-regression), if done, indicating which were pre-specified.	N/A
**RESULTS**			
**Study selection**	17	Give numbers of studies screened, assessed for eligibility, and included in the review, with reasons for exclusions at each stage, ideally with a flow diagram.	3.1 Search Results
**Study characteristics**	18	For each study, present characteristics for which data were extracted (e.g., study size, PICOS, follow-up period) and provide the citations.	SI Table
**Risk of bias within studies**	19	Present data on risk of bias of each study and, if available, any outcome level assessment (see item 12).	N/A
**Results of individual studies**	20	For all outcomes considered (benefits or harms), present, for each study: (a) simple summary data for each intervention group (b) effect estimates and confidence intervals, ideally with a forest plot.	N/A
**Synthesis of results**	21	Present results of each meta-analysis done, including confidence intervals and measures of consistency.	N/A
**Risk of bias across studies**	22	Present results of any assessment of risk of bias across studies (see Item 15).	N/A
**Additional analysis**	23	Give results of additional analyses, if done (e.g., sensitivity or subgroup analyses, meta-regression [see Item 16]).	N/A
**DISCUSSION**			
**Summary of evidence**	24	Summarize the main findings including the strength of evidence for each main outcome; consider their relevance to key groups (e.g., healthcare providers, users, and policy makers).	4. Discussion
**Limitations**	25	Discuss limitations at study and outcome level (e.g., risk of bias), and at review-level (e.g., incomplete retrieval of identified research, reporting bias).	4.4 Limitations
**Conclusions**	26	Provide a general interpretation of the results in the context of other evidence, and implications for future research.	5. Conclusions
**FUNDING**			
**Funding**	27	Describe sources of funding for the systematic review and other support (e.g., supply of data); role of funders for the systematic review.	Funding Statement

From: Moher D, Liberati A, Tetzlaff J, Altman DG, The PRISMA Group (2009). Preferred Reporting Items for Systematic Reviews and Meta-Analyses: The PRISMA Statement. PLoS Med 6(7): e1000097. doi:10.1371/journal.pmed1000097 . For more information, visit: **www.prisma-statement.org**.

SI 2: SEARCH STRATEGY

Table S2-1: Databases Included in Literature Search

Database Name	Description	Rationale	Database Type	Literature Types	Search Results
ASCE Research Library	Articles from ASCE journals, papers from conference proceedings, and e-books and standards.	Preliminary reviews showed that it contained many sources of complex systems analysis for water and wastewater service delivery.	Organization	Peer-reviewed	821
Carolina Digital Repository	Repository of University of North Carolina publications (student, thesis, etc..).	Includes documents from documents from the Gillings School of Global Public Health at UNC and The Water Institute at UNC, conveners of the Water & Health Conference, one of the largest annual WASH conferences.	Academic Institution	Peer-reviewed<br>Grey<br>Thesis	13
Deutsche Gesellschaft für Internationale Zusammenarbeit (GIZ) Library	Database of Deutsche Gesellschaft für Internationale Zusammenarbeit GmbH (GIZ), German development agency.	One of the larger and most innovative bilateral organizations working in the WASH sector worldwide. Also, open source resource.	Organization	Grey<br>Organizational<br>Open Source	109
DFID	Database of The Department for International Development (DFID), which manages the UK Government's aid programs.	A large funder of WASH projects worldwide and early adopter of systems approaches to development	Organization	Grey<br>Organizational<br>Open Source	1
EBSCO	Provider of 375 full-text databases for e-journals, magazine subscriptions,	One of the largest and most comprehensive databases covering WASH and systems research topics.	Provider	Peer-reviewed	4,129

	ebooks and discovery service for academic libraries, public libraries.				
ELDIS (Int'l development)	Eldis repository of the Institute of Development Studies managed by Knowledge, Impact and Policy team.	Database focused specifically on international development topics, including WASH, with a range of peer-reviewed and grey literature that is open source.	Provider	Grey<br>Peer-reviewed<br>Open Source	151
International Rescue Committee Library	Repository of The International Rescue Committee (IRC), a global humanitarian aid, relief, and development nongovernmental organization.	IRC is a large donor and implementer in the WASH sector, including in systems approaches to WASH.	Organization	Organizational	44
IRC WASH	Library of IRC WASH - formerly the WHO International Reference Center on Community Water Supply (abbreviated as I.R.C.).	Database consists of publications by IRC WASH and others. One of the largest repositories of WASH systems-focused work.	Organization	Organizational	1,357
Loughborough University Institutional Repository	Repository of Loughborough University publications (student, thesis, etc..).	Includes documents from Loughborough's Water, Engineering and Development Centre (WEDC), an innovative WASH research center and convener of a large annual conferences on innovative directions in WASH.	Academic Institution	Peer-reviewed<br>Thesis	27
OECD iLibrary	OECD iLibrary is the online library of the Organisation for Economic Cooperation and Development (OECD) featuring its books, papers	OECD is a large funder of innovative research and projects in the infrastructure services sector, including water supply and sanitation.	Organization	Grey<br>Organizational	13

	and statistics and is the gateway to OECD's analysis and data.				
PAIS International	International coverage of public affairs, public and social policies, and international relations. It includes two databases, PAIS International and PAIS Archive.	Provider of peer-reviewed-reviewed and grey literature from countries outside of North America and Europe, focused on international topics, including WASH.	Provider	Peer-reviewed Grey	518
ProQuest Dissertations and Theses	ProQuest for Dissertations	Used to specifically search for dissertations on WASH systems topics which may have not been disseminated through peer-reviewed reviewed or grey sources.	Provider	Thesis	376
SciELO	SciELO is a bibliographic database, digital library, and cooperative electronic publishing model of open access journals. Primarily with non-English publications	Large database representing non-English language publications, including non-WASH topics, which may not appear in other journals or databases.	Provider	Peer-reviewed	244
SuSanA	Library, project database and discussion forum for the Sustainable Sanitation Alliance, an informal network of people and organizations	Organizational and grey literature specifically focused on emerging sanitation topics, both from the SuSanA and its members.	Organization	Grey Organizational	46
University of South Florida Repository	Repository of USF publications (student, thesis, etc..).	USF is home to the International Development Engineering Program, a teaching and research center focused on new directions and approaches to international development.	Academic Institution	Peer-reviewed Thesis	24

Water and Sanitation Program (WSP) Library	A multi-donor partnership, part of the World Bank Group's Water Global Practice focused on water and sanitation.	Leading producer of research and practice in water and sanitation topics. Early adopter of systems discourses in WASH.	Organization	Peer-reviewed Grey Organizational	105
Water Supply & Sanitation Collaborative Council (WSSCC) Resources	A global, multi-stakeholder membership and partnership organization that works with poor people, organizations, governments and local entrepreneurs to improve sanitation and hygiene at scale.	Similar to WSP, a large producer of research and practice documentation for WASH, including innovative approaches to service delivery.	Organization	Grey	35
Web of Science	Premier multidisciplinary index covering the journal literature of the sciences.	Focus on multidisciplinary research on WASH topics to augment engineering- and public health-focused databases included in the literature review	Provider	Peer-reviewed	379
World Bank eLibrary	Collection of World Bank publications since the 1990s.	World Bank is one of the largest legacy funders of WASH projects worldwide. This database contains all project reports and evaluations on WASH projects from the bank that have been made public.	Organization	Organizational	670

Table S2-2: WASH and Systems Keywords and Search String Formula

WASH Keywords	Systems Keywords	Search String
drinking water<br>hand washing<br>hygiene<br>latrine<br>sanitation<br>toilet<br>WASH<br>water access<br>water management<br>water quality<br>water resource<br>water service<br>water source<br>water supply<br>water use	actors<br>adapt<br>alliance<br>approach<br>coalition<br>complex<br>connect<br>dynamics<br>emergent<br>factors<br>feedback<br>holistic<br>inter<br>network<br>scenario<br>social<br>stakeholder<br>system	"drinking water" OR "hand washing" OR "hygiene" OR "latrine" OR "sanitation" OR "toilet" OR "WASH" OR "water access" OR "water management" OR "water quality" OR "water resource" OR "water service" OR "water source" OR "water supply" OR "water use" OR<br><br>AND<br><br>"actors" OR "adapt" OR "alliance" OR "approach" OR "coalition" OR "complex" OR "connect" OR "dynamics" OR "emergent" OR "factors" OR "feedback" OR "holistic" OR "inter" OR "network" OR "scenario" OR "social" OR "stakeholder" OR "system" OR adapt* OR complex* OR connect* OR dynam* OR inter* OR system* OR

Table S2-3: Initial Screening Exclusion Terms and Criteria

Exclusion Term	Exclusion Criteria
Non-WASH	Study is not applicable to any aspect of the Water, Sanitation & Hygiene Sector.
Water Treatment	Study of water treatment or water quality including processes, technologies, or microbiology (e.g. evaluation of a ceramic filter design for use in rural communities).
Wastewater Treatment	Study of wastewater treatment or water quality including processes, technologies, or microbiology (e.g. evaluation of an eco-san toilet design).
Hydrology	Study of physical hydrology of water resources including drinking water, wastewater and non-consumption water.
Water Resource Management	Study related to the management of water, land and other natural resources for non-consumptive use including irrigation, stream by flows or hydropower.
Public Health (WASH)	Study focused on public health impacts of a WASH program / intervention but does not investigate multiple factors or attributes of that impact.
Meta-Analysis	Study represented reviews or a meta-analysis of existing literature on a WASH topic. Full text documents for references labeled as meta-analysis were obtained and searched for additional studies which may meet eligibility criteria.
Non-Systems	Study is related to an aspect of WASH but does not describe an analysis or process that investigates two or more aspects, factors or attributes of the WASH context. (i.e. post-project report evaluating the effectiveness of a sanitation intervention program, cost-effectiveness analysis).
Descriptive Statistics	A study which analyzes the correlations between multiple variables against a chosen WASH outcome, but which is decidedly not 'systematic' in nature

SI 3: WORKSHEET AND CODING FORM

Table S3-1: Coding Form

Attribute	Descriptors	Evaluation Criteria
Methodology	Method[2]	Stated name of method of analysis or approach employed by the study (open response) and open-coding of methodologies
	Analytical Complexity[1]	Low - Non-computational tasks can be completed without specialized knowledge or training<br>Medium - Some specific knowledge or training is required to complete computational tasks<br>High - Process requires a high level of specialized knowledge or training to conduct the analysis
	Interactions[1]	Does the method explicitly consider interactions among factors? (Yes / No)
	Sources of data[2]	Open coding of source of data for the study (e.g. surveys, interviews, focus groups, observations, etc.)
	Factors[2]	Open coding of factors included in the study and count (e.g. finance, hardware, regulations)
	Method Application[1]	Analysis – Application of an established analytical method, presented without a broader theory on how it should be applied, or steps for applying it (e.g. system dynamics modeling of rural water functionality)<br>Tool – A discrete, standalone activity or analysis presented with sufficient detail to be readily replicated; or, an analytical program or software (e.g. asset management tool for water utilities)<br>Framework – A guiding outline of activities, analyses or procedures for applying the method (e.g. Sanitation Cityscape Conceptual Framework)<br>Approach – A theoretical or conceptual construct, without discrete steps for implementation of the method (e.g. Livelihoods Approach)
Scope	Aspects of WASH[1]	Water, Sanitation and/or Hygiene
	Outcome Scope[2]	Emergent coding of study outcomes or dependent variable (e.g. behavior change, service sustainability)
	Context[1]	Rural / Urban
		Local (incl community), City, Regional (incl District), National, Sector
Application	Project Application[1]	Assessment of what stage, if any, the study was implemented in relation to a WASH infrastructure or services project:<br>Planning – Used to plan future WASH service delivery project;<br>Implementation – Method used to guide the implementation of a WASH project<br>Evaluation – Post-project assessment of outcomes<br>Case Study – Standalone analysis of a specific case(s), not related to a WASH project<br>None – No project application conducted

	**Location[1]**	Name and count of geographic locations where study was applied
	**Reporting of Impacts[2]**	Stated outcomes or effects on WASH services that occurred as a result of the application of the study (if any)

Secondary Screening Criteria Coding Form including Attributes, Descriptors and Evaluation Criteria. Notes: 1: a-priori, deductive criteria; 2: open or emergent criteria

Table SI3-2: Literature Search Worksheet

Document Information								Method						Scope				Application					Resources		
**Document ID**	**Database**	**Title**	**Author(s)**	**Date**	**Publication**	**Document Type**	**Open Access**	**Method Analysis**	**Analytical Complexity**	**Interaction Analysis**	**Data Source Analysis**	**Factors**	**Method Application**	**Aspects of WASH**	**Rural / Urban**	**Context**	**Outcome Scope**	**Project Application**	**Location**	**Location Region**	**Location Count**	**Study Impacts**	**Time**	**Human Resources**	**Cost**
2	Hand Search	Understanding handpump sustainability: Determinants of Rural water source functionality in the Greater Afram Plains region of Ghana	M. Fisher, K. Shields, T. Chan, E. Christenson, R. Cronk, H. Leker, D. Samani, P. Apoya, A. Lutz, J. Bartram	2015	Water Resources Research	Peer	X	Bayesian Networks	High	Yes	Secondary Data	Social<br>Financial<br>Technical<br>Environmental<br>Context	Analysis	Water	Rural	Regional	Sustainability	Case Study	Greater Afram Plains region of Ghana	West Africa	1				
5	Hand Search	Assessent of Sustainability of Rural Water, Sanitation, and Hygiene Interventions in Rwanda	M. Malik, L. Karangwa, A. Muzula, J. Sano, J.M. Rutaganda, G. Musabyimana	2016	RWSN	Peer	X	Composite Scoring	High	No	Surveys<br>Interviews<br>Secondary Data	Institutional<br>Social<br>Financial<br>Technical	Tool	WASH	Rural	Regional	Sustainability	Evaluation	Rural Rwanda	East Africa	1		4 months		$85,000
6	Hand Search	Against the Current: How to Shape an Enabling Environment for Sustainable Water Service Delivery in Nigeria	H. Hima, C. Santibanez, H. Kida, K. Bain	2015	Global Delivery Initiative	Grey	X	Qualitative Data Analysis	High	No	Interviews<br>Secondary Data<br>Primary Data<br>Observation<br>Focus Groups	Governance<br>Institutional<br>Capacity<br>Services<br>Social<br>Financial<br>Political<br>Coordination	Analysis	Water	Rural<br>Urban	Regional<br>National<br>Sector	Sustainability	Evaluation	Nigeria	West Africa	1				
7	Hand Search	Rural Water Supply and Sanitation in Developing Countries	S. Bhattarai and M. Starkle	2005	International Symposium on the Analytic Hierarchy Process	Peer	X	AHP<br>Multi-Criteria Decision Analysis	Medium	No	Secondary data<br>Primary Data<br>Surveys<br>Expert Opinion	Institutional<br>Social<br>Environmental<br>Financial<br>Technical	Analysis	Water<br>Sanitation	Rural	Local<br>Regional	Sustainability	Planning	Nepal	South Asia	1				
8	Hand Search	Analytical Heirarchy Process for Rural Micro Project Sustainability Monitoring in Nepal	S. Bhattarai and B. Adhikari	2005	International Symposium on the Analytic Hierarchy Process	Peer	X	AHP<br>Multi-Criteria Decision Analysis	Medium	No	Expert Opinion<br>Interviews<br>Focus Groups<br>Observation	Technical<br>Institutional<br>Social<br>Environmental<br>Financial<br>Behavioral	Tool	Water<br>Sanitation<br>Hygiene	Rural<br>Urban	Local	Sustainability	Evaluation	13 districts of Nepal (4 terai, 9 hill and 3 urban projects).	South Asia	13				
9	Hand Search	Building environments to support sustainability of improved sanitation behaviours at scale: levers of change in East Asia	N. Mukherjee	2016	Practical Action	Grey	X	Framework<br>Theory of Change	High	No	Secondary Data	Institutional<br>Financial<br>Capacity<br>Social<br>Monitoring	Framework	Sanitation	Rural	National<br>Sector	Sustainability	Case Study	Lao<br>Vietnam<br>Indonesia	South Asia	3				
10	Hand Search	Going beyond mason training: enabling, facilitating, and engaging Rural sanitation markets for the base of the pyramid	J. Dumpert and E. Perez	2015	Waterlines	Peer		Market Based-Approach Framework	Low	No	Secondary Data	Policy<br>Social<br>Infrastructure<br>Financial<br>Coordination<br>Capacity<br>Monitoring<br>Private Sector	Framework	Sanitation	Rural	National<br>Sector	Sustainability	Evaluation	Cambodia<br>Indonesia<br>Lao<br>Philippines<br>Vietnam<br>Peru<br>Ethiopia<br>Kenya<br>Tanzania<br>Uganda<br>Bangladesh<br>India<br>Pakistan	South Asia<br>Southeast Asia<br>East Africa<br>Central Asia	22				
14	Hand Search	Using Causal Loop Diagramming to Explore the Drivers of the Sustained Functionality of Rural Water Services in Timor-Leste	K. Neely and J. Walters	2016	Sustainability Journal	Peer	X	System Dynamics<br>Causal Loop Diagramming	High	Yes	Surveys<br>Interviews<br>Observation<br>Expert opinion	Donors<br>NGOs<br>Technical<br>O&M<br>Financial<br>Social	Analysis	Water	Rural	Local	Sustainability	Case Study	Timor-Leste	Southeast Asia	1				
15	Hand Search	A Framework to Assess Sustainability of Community-based Water Projects Using Multi-Criteria Analysis	K. Panthi and S. Bhattarai	2008	First International Conference on Construction In Developing Countries (ICCIDC–I)	Peer	X	AHP<br>Multi-Criteria Decision Analysis	Medium	No	Observation<br>Focus Groups<br>Interviews<br>Primary Data	Technical<br>Environmental<br>Social<br>Institutional<br>Financial	Tool<br>Framework	Water	Rural	Local	Sustainability	Case Study	16 communities in Nepal	South Asia	1				
19	Hand Search	A planning-oriented sustainabiilty assessment framework (POSAF) for peri-Urban water management in developing countries	N. Starkl, M. Brunner, E. Lopez, J. Martinez-Ruiz	2013	Water Research	Peer		Framework<br>Social Network Analysis<br>Multi-Criteria Decision Analysis<br>AHP<br>Statistics	High	No	Focus Groups<br>Interviews<br>Surveys	Technical<br>Economic<br>Networks<br>Environmental<br>Institutional<br>Financial<br>Users	Tool<br>Framework	Water	Urban	City	Sustainability	Planning	Xochmilco, Mexico	Central America	1	Coordination			
21	Hand Search	Management of Rural water services in Nicaragua: a systemic network approach to evaluating stakeholder alignment	J. Walters and A. Javernick-Will	2015	International Journal of Sustainable Development & World Ecology	Peer		Qualitative Data Analysis<br>Social Network Analysis<br>System Dynamics	High	Yes	Focus Groups	Technical<br>Management<br>Community<br>Social<br>Governance<br>Technical<br>Environmental<br>Functionality<br>Financial<br>Coordination	Analysis	Water	Rural	Local	Sustainability	Case Study	Terrabona, Nicaragua	Central America	1		Stakeholder Focus group sessions lasting between 2 and 3 hours		
22	Hand Search	Long-Term Functionality of Rural Water Services in Developing Countries: A System Dynamics Approach to Understanding the Dynamic Interaction of Factors	J. Walters and A. Javernick-Will	2015	Environmental Science & Technology	Peer		System Dynamics	High	Yes	Secondary Data<br>Expert Opinion	Functionality<br>Governance<br>Financial<br>Technical<br>Environmental<br>Energy<br>Community Management	Analysis	Water	Rural	Local	Sustainability	Case Study							
23	Hand Search	Exploring the use of social network analysis to inform exit strategies for Rural water and sanitation NGOs	J. Walters	2016	Engineering Project Organization Journal	Peer		Social Network Analysis	High	Yes	Interviews<br>Surveys	Coordination<br>NGOs<br>Networks<br>Technical<br>Functionality	Analysis	Water<br>Sanitation	Rural	Local	Sustainability	Case Study	Darío, Nicaragua.	Central America	1				
25	Hand Search	Planning Rural water services in Nicaragua: A systems-based analysis of impact factors using graphical modeling	J. Walters and P. Chinowsky	2016	Environmental Science & Policy	Peer		Graphic Modeling<br>Statistics<br>Network Analysis	High	Yes	Interviews<br>Observation<br>Primary Data	Governance<br>Community Management<br>Financial<br>Technical<br>Environmental<br>Energy<br>Functionality	Analysis	Water	Rural	Local	Access	Case Study	Rural communities in Darío, and Terrabona in Nicaragua	Central America	1				
27	Hand Search	Rethinking Sustainabilty, Scaling Up, and Enabling Environment: A Framework for Their Implementation in Drinking Water Supply	U. Amjad, E. Ojomo, K. Downs, R. Cronk, J. Bartram	2015	Water	Peer	X	Framework	Low	No	Secondary Data	Implementation<br>Functionality	Analysis	Water	Rural<br>Urban	Local	Sustainability	Case Study							

29	Hand Search	The Political Economy of Sanitation: How can we increase investment and imporove service for the poor	S. Garbarino, J. Holland, S. Brook, K. Caplan, A. Shankland	2011	WSP	Org	X	Framework	Medium	No	Secondary Data<br>Interviews<br>Focus Groups	Social<br>Political<br>Economic	Framework	Sanitation	Rural<br>Urban	National<br>Sector	Sustainability	Evaluation	Brazil<br>India<br>Indonesia<br>Senegal	South America<br>Southeast Asia<br>South Asia<br>West Africa	4				
30	Hand Search	Strengthening Enabling Environment for WASH	D. Tsetse, E. Kouassi-Komlan, C. Scharp, G. Hutton	2016	UNICEF	Org	X	Framework<br>Theory of Change	Medium	No	Expert Opinion<br>Primary Data<br>Secondary Data<br>Interviews	Institutional<br>Financial<br>Governance<br>Monitoring<br>Infrastructure<br>Planning<br>Services	Framework<br>Approach<br>Tool	WASH	Rural<br>Urban	National<br>Sector	Sustainability								

Document Information								Method						Scope				Application					Resources		
**Document ID**	**Database**	**Title**	**Author(s)**	**Date**	**Publication**	**Document Type**	**Open Access**	**Method Analysis**	**Analytical Complexity**	**Interaction Analysis**	**Data Source Analysis**	**Factors**	**Method Application**	**Aspects of WASH**	**Rural / Urban**	**Context**	**Outcome Scope**	**Project Application**	**Location**	**Location Region**	**Location Count**	**Study Impacts**	**Time**	**Human Resources**	**Cost**
32	Hand Search	Whole system change: capturing the change process in the Ghana Rural water sub-sector	H. Lockwood and V. Duti	2015	IRC WASH	Org	X	Collective Impact<br>Whole Systems Approach<br>Service Delivery Approach	Low	No	Primary Data<br>Secondary Data<br>Focus Groups	Institutional<br>Financial<br>Coordination<br>Governance<br>Regulations	Approach<br>Framework	Water	Rural	Local<br>National<br>Sector	Sustainability	Planning<br>Implementing<br>Evaluation	Ghana	West Africa	1	Policy<br>Services	The long time frame – **at least six years and potentially up to ten years**	The backbone support was provided by a mix of IRC staff assigned to the project and Triple-S project staff hired specifically for the project and hosted by CWSA. A much larger number of people also provided support to the project on a part-time basis, including both Ghana-	from 2009 to the end of May 2013 the total costs of running Triple-S in Ghana for IRC have been US$ 3,469,924. This price tag includes all international staff support, but does not include other investments made by IRC Ghana through activities under WASHCost and the Tripartite Partnership
37	Hand Search	A qualitative comparative analysis of well-managed school sanitation in Bangladesh	C. Chatterley, A. Javernick-Will, K. Linden, K. Alam, L. Bottinelli, M. Venkatesh	2013	BioMed Central	Peer	X	QCA<br>Qualitative	High	No	Interviews<br>Focus Groups<br>Observation	Services<br>Technical<br>Community<br>Governance<br>User Committees<br>O&M	Analysis	Sanitation	Rural	Local	Sustainability	Case Study	16 case schools in Meherpur, Bangladesh	South Asia	1				
41	Hand Search	Community Assessment Model for Sustainable Municipal Sanitation Services in low income communities	G. Louis and M. Bouabid	2004	International Council on Systems Engineering	Peer		Framework<br>Composite Scoring	Medium	No	Expert Opinion<br>Secondary Data	Institutional<br>Capacity<br>Technical<br>Economic<br>Financial<br>Energy<br>Environmental<br>Cultural	Tool	Sanitation		Local	Sustainability	Evaluation							
42	Hand Search	A decision model for selecting sustainable drinking water supply and greywater reuse systems for developing communities with a case study in Cimahi, Indonesia	J. Henriques and G. Louis	2010	Journal of Environmental Management	Peer	X	Composite Scoring	High	Yes	Interviews<br>Secondary Data<br>Observation	Functionality<br>Institutional<br>Capacity<br>Technical<br>Economic<br>Financial<br>Energy<br>Environmental<br>Social<br>Cultural	Tool	Water	Urban	Local	Sustainability	Planning<br>Case Study	Cimahi, Indonesia	Southeast Asia	1				
43	Hand Search	Introducing SaniFOAM: A Framework to Analyze Sanitation Behaviors to Design Effective Sanitation Programs	J. Devine	2009	World Bank	Grey	X	Framework	Medium	No	Secondary Data	Technical<br>Financial<br>Behavioral<br>Regulations<br>Social	Approach<br>Framework	Sanitation	Rural<br>Urban	Local<br>City	Sustainability	Planning<br>Implementation	Tanzania (10 districts), Indonesia (East Java), and India (in two states–Madhya Pradesh and Himachal Pradesh).	East Africa<br>South Asia<br>Southeast Asia	3				
44	Hand Search	A Suite of Tools to Support a Systems-Based Approach to Sustainable Management of Water Service Delivery	K. Lemme, K. Latham, K. Kugler	2016	Water For People	Org	X	Composite Scoring	Medium	No	Interviews<br>Primary data<br>Secondary Data	Services<br>Institutional<br>Financial<br>Environmental<br>Management<br>O&M<br>Monitoring	Tool<br>Framework	Water	Rural<br>Urban	Regional	Sustainability	Implementation<br>Evaluation<br>Case Study	Applied in 30 districts in 5 countries. Results presented from; Kamwenge District, Uganda Praganas District, India (District name?), Peru (rural district), Malawi Sagar Island, India Rulindo, Rwanda San Rafael del Norte, Nicaragua El Negrito, Honduras	East Africa<br>South Asia<br>Central America<br>South America	30	Financial			
47	Hand Search	Synthesis of Four Country Enabling Environment Assessments for Scaling Up Handwashing Programs	F. Rosenweig	2008	World Bank	Grey	X	Composite Scoring	Medium	No	Expert Opinions<br>Observations<br>Secondary Data	Policy<br>Coordination<br>Institutional<br>Capacity<br>Technical<br>Financial<br>Implementation<br>Monitoring	Tool	Sanitation<br>Hygiene	Rural	National	Behavior Change	Planning<br>Implementation<br>Evaluation	Peru<br>Senegal<br>Tanzania<br>Vietnam	South America<br>West Africa<br>East Africa<br>Southeast Asia	4	Behavior Change<br>Uptake			
50	Hand Search	Governence into Functionality (GIFT) Tool	S. Ogden	2013	CARE	Grey	X	Composite Scoring	Medium	No	Local Expert Opinion	Governance<br>Financial<br>Management<br>Users<br>Functionality	Tool	Water<br>Sanitation		Local	Sustainability	Evaluation						3-10 community members	
52	Hand Search	Methodology for Participatory Assessment (MPA)	R. Dayal, C. van Wijk, N. Mukherjee	2000	WSP, IRC WASH	Org	X	Framework<br>Statistics<br>Participatory<br>Composite Scoring	High	No	Surveys<br>Interviews<br>Observation<br>Primary Data<br>Secondary Data	Gender<br>Technical<br>Social<br>Hygiene<br>Participation<br>Institutional<br>Users<br>Social<br>Community<br>Policy	Approach<br>Tool<br>Framework	Water<br>Sanitation		Local	Sustainability	Planning<br>Evaluation	Eighteen assessments across 18 communities in 15 countries. (no mention of locations)	(no mention of locations in report)	18				
53	Hand Search	A model for assessing sustainability A web-based decision support system for predicting and evaluating sustainability (Toppes)	S. Takougan and J. Wethe	2016	Arima Journal	Peer	X	Composite Scoring<br>Multi-Criteria Decision Analysis	Medium	No	Expert Opinion	Socioeconomic<br>Services<br>Environmental<br>Technical<br>Financial<br>O&M<br>Institutional	Tool<br>Framework	Water	Rural	Local<br>Regional	Sustainability	Evaluation	Greater Afram Plains region of Ghana	West Africa	1				
54	Hand Search	The SIASAR Initiative: An Information System for More Sustainable Rural Water and Sanitation Services	L. Pena, D. Michaud, J. Biau	2013	World Bank	Grey	X	SIASAR<br>Composite Scoring	Low	No	Observation<br>Primary Data<br>Interviews<br>Expert Opinion	Access<br>Management<br>Functionality<br>Hygiene<br>Health<br>Financial<br>O&M	Tool	Water<br>Sanitation	Rural	Local<br>Regional	Sustainability	Evaluation	Nicaragua<br>Panama<br>Honduras	Central America	3				

56	Hand Search	Addressing WaSH challenges in Pacific Island Countries: A participatory marketing systems mapping approach to empower informal settlement community action	S. Saunders, D. Barrington, S. Sridharan, S. Meo, W. Hadwen, K. Shields, R. Souter, J. Bartram	2016	Habitat International	Peer	X	Participatory Systems Mapping	Low	No	Interviews Observation	Technical Context Networks Private Sector Socioeconomic User Committees	Tool	Water Sanitation	Urban	Local	Access	Case Study	Suva City, Fiji	Oceania	1		chairman of the informal settlement invited the research team to visit the community over a two-week period to specifically undertake the systems mapping activities.	Two-person research team	

Document Information								Method						Scope				Application					Resources		
**Document ID**	**Database**	**Title**	**Author(s)**	**Date**	**Publication**	**Document Type**	**Open Access**	**Method Analysis**	**Analytical Complexity**	**Interaction Analysis**	**Data Source Analysis**	**Factors**	**Method Application**	**Aspects of WASH**	**Rural / Urban**	**Context**	**Outcome Scope**	**Project Application**	**Location**	**Location Region**	**Location Count**	**Study Impacts**	**Time**	**Human Resources**	**Cost**
57	Hand Search	Water and Sanitation Hygiene Bottleneck Analysis Tool (WASH BAT 2.0) User Manual	UNICEF (Uncredited)	2016	WASH BAT	Grey	X	Composite Scoring	Medium	No	Local Expert Opinion	Regulations<br>Financial<br>Private Sector<br>O&M<br>Social<br>Environmental	Tool<br>Framework	WASH	Rural<br>Urban	National	Sustainability<br>Access	Evaluation<br>Planning							
59	Hand Search	Assessing Women's Negative Sanitation Experiences and Concerns: The Development of a Novel Sanitation Insecurity Measure	B. Caruso, T. Clasen, K. Youn, H. Cooper, C. Hadley, R. Haardörfer	2017	International Journal of Environmental Research and Public Health	Peer	X	Framework<br>Statistics	High	Yes	Interviews<br>Focus Groups<br>Surveys	Social<br>Technical<br>Technical<br>Community<br>Behavioral	Tool	Sanitation	Rural	Local	Access	Case Study	Puri district, Odisha, India	South Asia	1				
60	Hand Search	WASH Sustainability Index Tool (SIT) Comparative Analysis	J. Annis and L. Moreland	2015	WASH Plus	Org	X	Composite Scoring	High	No	Interviews<br>Focus Groups<br>Secondary Data	Institutional<br>Management<br>Financial<br>Environmental<br>Technical	Tool	WASH	Rural<br>Urban	National	Sustainability	Evaluation	Burkina Faso<br>Dominican Republic<br>Ethiopia<br>Ghana<br>Kenya<br>Liberia<br>Niger<br>Philippines<br>Tanzania	East Africa<br>Caribbean<br>West Africa<br>Southeast Asia	9	Uptake	2-6 Months		$15-150k<br>Avg - $50k / country
63	Hand Search	Sanitation Monitoring Toolkit	UNICEF (Uncredited)	2014	UNICEF	Org	X	Composite Scoring<br>Framework	Medium	No	Secondary Data<br>Expert Opinion<br>Interviews	Financial<br>Policy<br>Planning<br>Social<br>O&M<br>Institutional<br>Private Sector<br>Coordination	Tool<br>Framework	Sanitation	Rural	Local<br>Regional<br>National	Access<br>Sustainability	Evaluation							
64	Hand Search	Modeling the Complexities of Water, Hygiene, and Health in Limpopo Province, South Africa	J. Mellor, J. Smith, G. Learmonth, V. Netshandama, R. Dillingha	2012	Environmental Science & Technology	Peer	X	System Dynamics	High	Yes	Secondary Data	Environmental<br>Technical<br>Technical	Analysis	Water	Rural	Local	Health	Case Study	(2) communities in South Africa	South Asia	2				
65	Hand Search	Developing A Systems Understanding of Rural Water Supply in Timor-Leste	K. Neely and J. Walters	2015	Deaking University	Peer	X	Social Network Analysis<br>Causal Loop Diagramming	High	Yes	Interviews<br>Observation<br>Focus Groups	Community<br>Networks<br>Management<br>NGOs<br>Coordination<br>Social<br>Economic<br>Environmental<br>O&M	Analysis<br>Approach	Water	Rural	Local	Access<br>Sustainability	Case Study	(5) Rural villages Timor-Leste	Southeast Asia	5				
66	Hand Search	A systems approach to sustainable water operation & maintenance in Uganda	N. Mary	2016	RWSN	Peer	X	Market-Based approach	Low	Yes		O&M<br>Private Sector<br>Governance<br>Regulations<br>User Committees	Tool	Water	Rural	Regional	Sustainability	Planning<br>Evaluation	Bugiri and Namayingo districts, Uganda	East Africa	2	Services			
70	Hand Search	Decision-making on shared sanitation in the informal settlements of Kisumu, Kenya	S. Simiyua, M. Swilling, S. Cairncross	2017	International Journal of Environmental Health Research	Peer		Qualitative Data Analysis	High	No	Interviews	Financial<br>Services<br>O&M<br>Socioeconomic	Analysis	Sanitation	Urban	Local	Sustainability	Case Study	Kisumu (informal settlements) Kenya	East Africa	1				
72	Hand Search	Tipping Points in Community-Based Management	G. Guerrero	2016	TU Delft	Peer	X	System Dynamics<br>Causal Loop Diagramming	High	Yes	Interviews<br>Secondary Data	Policy<br>Management<br>Community<br>Financial<br>Environmental<br>Access	Tool	Water	Rural	Regional	Sustainability	Case Study	Kabarole, Uganda	East Africa	1				
73	Hand Search	Implementing sustainable water and sanitation projects in Rural, developing communities	R. Barnes, N. Ashbolt, D. Roser, P. Brown	2014	Waterlines	Peer	X	Qualitative Data Analysis	Medium	No	Interviews	Community<br>Planning<br>Social<br>Implementation<br>Technical<br>Governance	Analysis	Water<br>Sanitation	Rural	Local	Sustainability	Case Study							
74	Hand Search	The Role of Learning in Water Supply Systems: Analysing the Spread of Policies in Rural Uganda using Memetics	F. Knipschild	2016	TU Delft	Peer	X	System Dynamics<br>Agent Based Modeling	High	Yes	Secondary Data	Capacity<br>Financial<br>Functionality<br>Coordination<br>Education<br>Technical<br>O&M	Tool	Water	Rural	Regional<br>National	Sustainability	Case Study	Uganda	East Africa	1				
77	Hand Search	Revealing causal pathways to sustainable water service delivery using fsQCA	K. Gasparro and J. Walters	2017	Journal of Water, Sanitation and Hygiene for Development	Peer		Qualitative Comparative Analysis	High	No	Secondary Data<br>Interviews	Community<br>Technical<br>User Committees<br>Financial<br>Coordination<br>Education	Analysis	Water	Rural	Local	Sustainability	Case Study	26 projects implemented by EWB across multiple countries		26				
78	Hand Search	Improving the robustness of financial and economic analysis of sanitation systems	J. Parkinson and S. Blume	2010	IRC WASH Symposium 2010: Pumps, Pipes and Promises	Org	X	Financial Analysis	Low	No	Secondary Data	Technical<br>Agriculture<br>Financial<br>Health<br>Implementation<br>Environmental<br>Economic	Analysis	Sanitation		Local	Sustainability	Planning<br>Case Study	Uganda<br>South Africa<br>Burkina Faso	East Africa<br>South Africa<br>West Africa	3				
80	Hand Search	Towards systemic change in urban sanitation	G. Galli, C. Nothomb, E. Baetings	2014	IRC WASH	Org	X	Whole Systems Approach<br>Service Delivery Approach<br>Shit Flow Diagrams<br>Theory of Change	Medium	No	Focus groups<br>Interviews<br>Primary data<br>Secondary data	Financial<br>Institutional<br>Environmental<br>Technical<br>Social	Approach<br>Framework	Sanitation	Urban	Local<br>Regional<br>National	Sustainability	Planning<br>Implementing	Ghana<br>Uganda	West Africa<br>East Africa	2	Users<br>Services			
81	Hand Search	The Triple-S Theory of Change	T. Schouten and P. Moriarty	2013	IRC WASH	Org	X	Framework<br>Service Delivery Approach<br>Theory of Change	Low	No	Secondary Data	Political<br>Coordination<br>Institutional<br>Monitoring<br>Capacity<br>Policy<br>Financial<br>Planning	Approach	Water	Rural	Local<br>Regional<br>National<br>Sector	Sustainability	Planning<br>Implementing	Ghana<br>Uganda<br>Mozambique<br>Burkina Faso	East Africa<br>West Africa	4				
82	Hand Search	Systems Approach to Climate, Water, and Diarrhea in Hubli-Dharwad, India	J. Mellor, E. Kumpel, A. Ercumen, J. Zimmerman	2016	Environmental Science & Technology	Peer		Agent Based Modeling	High	Yes	Primary data<br>Secondary data	Climate<br>Technical<br>Environmental<br>Health<br>Services	Tool<br>Framework	Water	Urban	City	Health	Case Study	Hubli-Dharwad in Karnataka, India	South Asia	1				

Document Information								Method						Scope				Application					Resources		
Document ID	Database	Title	Author(s)	Date	Publication	Document Type	Open Access	Method Analysis	Analytical Complexity	Interaction Analysis	Data Source Analysis	Factors	Method Application	Aspects of WASH	Rural / Urban	Context	Outcome Scope	Project Application	Location	Location Region	Location Count	Study Impacts	Time	Human Resources	Cost
83	Hand Search	Consolidation, Improvement and Expansion of the Rural Water and Sanitation Information System (SIASAR)	A. Serrano, C. Fenwick, D. Sobel, G. Crespo, M. Gonzalez, C. Borja-Vega	2017	World Bank	Grey	X	SIASAR Composite Scoring Framework	Low	No	Surveys	Services<br>Technical<br>Regulations<br>O&M<br>Financial<br>Access<br>Hygiene<br>Capacity<br>Institutional	Tool<br>Framework	Water<br>Sanitation	Rural	Local<br>Regional<br>National	Sustainability	Planning<br>Implementing<br>Evaluation	Honduras<br>Nicaragua<br>Panama<br>Dominican Republic<br>Costa Rica<br>Oaxaca (Mexico)<br>Peru<br>Bolivia<br>Colombia<br>Paraguay	Central America<br>South America	10	Uptake	Without improved drinking water supply or service provider<br>1 -2<br>With improved drinking water supply and service provider<br>4 – 8 (varies according and complexity and distance to source)<br>Technical assistance provider<br>1		The total estimated cost of the SIASAR regional initiative from FY14 to date is US$2.69 million,39 of which the Bank provided approximately US$1.83 million from Trust Fund (TF)40 resources.41. Recurring costs for activities such as the General Assembly (average cost
84	Hand Search	Sustainable Services Checklist	Water For People (Uncredited)	2018	Water For People	Org		Composite Scoring	Low	No	Interviews<br>Secondary Data	Institutional<br>Financial<br>Management<br>Monitoring<br>O&M<br>Environmental	Tool	Water<br>Sanitation	Rural<br>Urban	Regional	Sustainability	Evaluation	Arani, Bolivia<br>Arbieto, Bolivia<br>Cuchumuela, Bolivia<br>Pocona, Bolivia<br>San Benito, Bolivia<br>San Pedro, Bolivia<br>Tiraque, Bolivia<br>Villa Rivero, Bolivia<br>San Andres Sajcabaja, Guatemala<br>San Antonio Illotenango, Guatemala<br>San Bartolome, Guatemala<br>Santa Cruz del Quiche, Guatemala<br>Chinda, Honduras<br>El Negrito, Honduras<br>San Antonia, Honduras<br>La Concordia, Nicaragua<br>San Rafael del Norte, Nicaragua<br>Asuncion, Peru<br>Cascas, Peru	South America<br>Central America<br>East Africa<br>South Asia	26		TIMELINE<br>There is pre-work, interview, and post-work to complete the SSC.<br>Timing Activity<br>At least 2 weeks before the interview Country Program staff should share relevant background information with the interviewer. Background information could include	TIMELINE<br>There is pre-work, interview, and post-work to complete the SSC.<br>Timing Activity<br>At least 2 weeks before the interview Country Program staff should share relevant background information with the interviewer. Background information could include	
85	Hand Search	Priority Addressment Protocol: Understanding the Ability and Potential of Sanitation Systems to Address Priorities	A. Davis, A. Javernick-Will, S. Cook	2019	Environmental Science & technology	Peer		Framework<br>AHP	High	No	Interviews<br>Focus Groups<br>Primary Data	O&M<br>Technical<br>Social<br>Financial<br>Community	Framework	Sanitation	Urban	City	Sustainability	Evaluation	20 slum communities in India	South Asia	1				
86	Hand Search	DWA Sustainability Monitoring Framework – The Guide	Dutch Water Alliance, IRC (Uncredited)	2014	Dutch Water Alliance	Org	X	Composite Scoring	Low	No	Surveys<br>Focus Groups<br>Secondary Data	Financial<br>Institutional<br>Environmental<br>Technical<br>Social	Tool	WASH	Rural	Local<br>Regional	Sustainability	Evaluation	Uganda<br>Ghana	East Africa	2				
88	Hand Search	Characteristics of Stakeholder Networks Supporting Local Government Performance Improvements in Rural Water Supply: Cases from Ghana, Malawi, and Bolivia	D. McNicholl, A. McRobie, H. Cruickshank	2017	Water Alternatives	Peer		Social Network Analysis	High	Yes	Interviews	Networks<br>Institutional<br>Governance<br>Coordination	Analysis	Water	Rural	Local<br>Regional	Coordination	Case Study	(7) local governments in<br>(2) Ghana<br>(2) Malawi<br>(3) Bolivia	South America<br>East Africa<br>West Africa	7				
89	Hand Search	An evaluative framework for urban WASH sector functionality	S. Drabble, R. Renoug, J. Stokes	2018	Water and Sanitation for the Urban Poor	Org	X	Composite Scoring	Low	No	Workshop<br>Surveys	Monitoring<br>Coordination<br>Regulations<br>Financial<br>Capacity<br>Private Sector<br>Policy	Framework<br>Tool	WASH	Urban	National<br>Sector	Policy	Evaluation<br>Planning	Bangladesh<br>Ghana<br>Kenya<br>Madagascar<br>Mozambique<br>Zambia	South Asia<br>West Africa<br>East Africa	6	Coordination<br>Policy	Two-day long workshop		
90	Hand Search	The use of qualitative comparative analysis to identify pathways to successful and failed sanitation systems	A. Davis, A. Javernick-Will, S. Cook	2019	Science of the Total Environment	Peer		Qualitative Comparative Analysis	High	Yes	Interviews<br>Observations<br>Primary Data	Behavioral<br>Education<br>O&M<br>Community<br>Infrastructure<br>Governance<br>Planning<br>Financial	Analysis	Sanitation	Urban	Local	Functionality	Case Study	20 communities (i.e., cases) in Karnataka or Tamil Nadu, India were selected to ensure variability between outcomes and factors (i.e., causal conditions)	South Asia	20				
91	Hand Search	Building strong WASH systems for the SDGs: Understanding the WASH system and its building blocks	A. Huston and P. Moriarty	2018	IRC WASH	Org	X	Building Blocks	Low	Yes	Expert Opinion<br>Secondary Data	Policy<br>Institutional<br>Infrastructure<br>Monitoring<br>Environmental<br>Regulations<br>Financial<br>Planning<br>Education<br>Health<br>Management	Tool<br>Framework	WASH	Rural<br>Urban	Local<br>Regional<br>National<br>Sector	Sustainability	Evaluation<br>Planning							
92	Hand Search	Sustainability Framework	R. Carter, V. Casey, E. Harvey	2011	WaterAid	Org	X	Framework	Low	No	Primary Data<br>Expert Opinion	Access<br>Infrastructure<br>Financial<br>Implementation<br>Monitoring<br>Environmental<br>Management<br>Donors	Framework	WASH	Rural<br>Urban	Local	Sustainability	Evaluation<br>Planning							
93	Hand Search	Sustainability Snapshot Indicator<br>Equity in Distribution Indicator	S. Sugden	2003	WaterAid	Org	X	Framework	Low	No	Primary Data<br>Surveys<br>Observations	Financial<br>Technical<br>O&M<br>Infrastructure<br>Access	Tool	Water	Rural	Local<br>Regional	Sustainability	Planning<br>Implementation	Malawi	East Africa	1	Uptake			

Document Information								Method						Scope				Application					Resources		
Document ID	Database	Title	Author(s)	Date	Publication	Document Type	Open Access	Method Analysis	Analytical Complexity	Interaction Analysis	Data Source Analysis	Factors	Method Application	Aspects of WASH	Rural / Urban	Context	Outcome Scope	Project Application	Location	Location Region	Location Count	Study Impacts	Time	Human Resources	Cost
94	Hand Search	Technology Applicability Framework (TAF) - Research Report	A. Olschewski and V. Casey	2013	Skat Foundation WaterAID	Org	X	Framework	Low	No	Primary Data Secondary Data Workshop Interviews Focus Groups	Financial Social Environmental Institutional Policy Technical Capacity Education	Tool Approach	Water Sanitation Hygiene	Rural Urban	Regional	Sustainability Access	Planning Implementation Evaluation	Burkina Faso Ghana Uganda	East Africa West Africa	3	Uptake	The TAF can be applied in a team of 2-3 persons per region.	Based on the experiences in three rounds of testing, it can be assumed that the application of the TAF requires around 3 days of preparation and 3 days in the field.	The TAF is a cost efficient tool. The costs for applying the TAF are estimated around US$ 3,000 per assessment of one technology in one district. A detailed breakdown of the costs for applying the TAF is presented in the Annex 6 of the TAF
95	Hand Search	Adapting Life-Cycle Thinking Tools to Evaluate Project Sustainability in International Water and Sanitation Development Work	J. McConville and J. Mihelcic	2007	Environmental Engineering Science	Peer	X	Lifecycle Analysis Composite Scoring Framework	Medium	No	Observation Survey Primary Data	Social Cultural Community Political Economic Environmental	Tool Framework	Water Sanitation	Rural	Local	Sustainability	Planning Evaluation	Zambougou-Fouta, Mali	West Africa	1				
96	Hand Search	Water and Sanitation Accountyability Forum; Organizational Evaluation of COCEPRADIL	F. Rojas and C. Chatterley	2011	Improve International	Grey	X	Framework Qualitative Data Analysis	Medium	No	Secondary Data Focus Groups Observation Interviews Surveys	NGOs Coordination Services Education Hygiene Technical O&M Environmental Community Management	Framework Approach	Water Sanitation	Rural	Local	Sustainability	Evaluation Case Study	Lempira, Honduras	Central America	1		For this pilot Accountability Forum, a week was given for preparation meetings, data collection and wrap-up. Though a week is not a substantial amount of time for a field-based study, independent evaluators and Forum participants felt that this was sufficient time to respond to the main		Based on the pilot test it is estimated that ratings using the Water for Life framework will cost between $US 15,000-20,000 per evaluation and require one week of field data collection and three to five months total including preparation and reporting. (Triple-S Summary)
97	Hand Search	Thousands of handpumps have been installed across sub-Saharan Africa over the past four decades to improve rural water supplies. However, multiple studies have raised concerns over the extent to which these sources are providing safe and adequate quantities of water post-construction. A number of factors could be causing these problems, either directly or indirectly. Understanding the interrelated nature of these factors and their inherent complexity is crucial if handpump failures are to be avoided in the future. This chapter demonstrates how some of this complexity can be	Elisabeth S. Liddle and Richard A. Fenner	2019	Systems Thinking in WASH	Grey		Causal Loop Diagrams	Medium	Yes	Interviews Expert Opinion	Technical O&M Capacity Environmental Services Institutional Financial Coordination	Framework Tool	Water	Rural	Local	Access Sustainability	Evaluation	Uganda	East Africa	1				
98	Hand Search	WASH services are influenced by complex stakeholder interactions that need to be understood and potentially influenced in order to improve service quality and sustainability. Social network analysis provides an opportunity for mapping and rigorously interpreting these stakeholder interactions in order to develop and monitor strategic interventions. Studies using social network analysis can be scaled to fit the scope of any project, and the visual nature of social networks helps to make findings accessible to a wide range of stakeholders. This chapter presents	Duncan McNicholl	2019	Systems Thinking in WASH	Grey		Social Network Analysis	High	Yes	Interviews	Coordination Institutions NGOs Networks	Tool	Water Sanitation	Rural Urban	Local Regional	Coordination	Planning Evaluating	Ghana Tajikistan Bolivia Uganda	East Africa Central Asia South America West Africa	4				

Document Information								Method						Scope				Application					Resources		
Document ID	Database	Title	Author(s)	Date	Publication	Document Type	Open Access	Method Analysis	Analytical Complexity	Interaction Analysis	Data Source Analysis	Factors	Method Application	Aspects of WASH	Rural / Urban	Context	Outcome Scope	Project Application	Location	Location Region	Location Count	Study Impacts	Time	Human Resources	Cost
100	Hand Search	Contemporary approaches to monitoring and evaluation (M&E) rely on using static indicators to assess a system that is fundamentally in flux. This chapter will look at the challenges of current approaches and provide insights into how to restructure the way we measure our impact on whole systems, rather than just individual processes. This chapter will explain how to adopt a system view of your operating environment, how to map that system to understand its interconnectedness and inherent complexity, and then how to identify the true drivers of the system to allow for the creation of a range of	Chris Brown	2019	Systems Thinking in WASH	Grey		Social Network Analysis Causal Loop Diagrams	High	Yes	Secondary Data	Monitoring Governence Institutional Management O&M	Tool Approach	Water	Rural	Regional	Sustainability	Evaluating	Tanzania	East Africa	1				
EBSCO AP 1	EBSCO	Towards sustainable water, sanitation and hygiene technology use in sub-Saharan Africa: the Learning Alliance approach.	S. Taylor, S. Asimah, R. Buamah, K. Nyarko, S. Sekuma, Y. Coulibaly, A. Wozuame, P. Jeffrey, H. Parker	2017	Water Policy	Peer	X	Collective Action	High	No	Interviews	Social Economic Environmental Institutional Technical Users	Approach	WASH	Rural		Access Sustainability	Evaluation	Burkina Faso Ghana Uganda	East Africa West Africa	3		3 years		
EBSCO AP 1033	EBSCO	A new approach to nationwide sanitation planning for developing countries: Case study of Indonesia.	S. Kerstens, M. Spiller, I. Leusbrock, G. Zeeman	2016	Science of the Total Environment	Peer		Framework GIS	High	Yes	Secondary Data Expert Opinions	Access Economic Financial Technical Infrastructure Policy	Tool Framework	Sanitation	Rural Urban	National	Access	Planning	Indonesia	Southeast Asia	1	Uptake			
EBSCO AP 14	EBSCO	A novel planning approach for the water, sanitation and hygiene (WaSH) sector: The use of object-oriented bayesian networks.	R. Gine-Garriga, D. Requejo, J. Molina, A. Perez-Foguet	2018	ScienceDirect	Peer	X	Bayesian Networks	High	Yes	Secondary Data Expert Opinion	Technical Capacity Financial Socioeconomic Access Functionality Coordination Management O&M	Tool	Water Sanitation Hygiene	Rural	Regional	Sustainability	Planning	21 Rural districts in Kenya	East Africa	1				
EBSCO AP 1619	EBSCO	Assessing poverty-alleviation outcomes of an enterprise-led approach to sanitation.	T. London and H. Esper	2014	Annals of the New York Academy of Sciences	Peer	X	Framework Qualitative Data Analysis Case Study	High	No	Secondary Data Interviews Observations	Access Community Networks Health Hygiene Economic	Analysis	Sanitation	Urban	City	Health	Evaluation Case Study	Nairobi, Kenya	East Africa	1				
EBSCO AP 165	EBSCO	A Holistic Concept to Design Optimal Water Supply Infrastructures for Informal Settlements Using Remote Sensing Data.	L. Rausch, J. Friesen, L. Altherr, M. Meck, P. Pelz	2018	Remote Sensing	Peer	X	Mathematical optimization Remote sensing	High	Yes	Secondary data	Context Financial Technical	Tool	Water	Urban	Local	Access	Planning	Two slum communities in Dhaka, Bangladesh, Dar es Salaam, Tanzania	East Africa South Asia	1				
EBSCO AP 17	EBSCO	Drinking Water Supply, Sanitation, and Hygiene Promotion Interventions in Two Slum Communities in Central Uganda.	D. Musoke, R. Ndejjo, A. Halage, S. Kasasa, J. Ssempebwa, D. Carpenter	2018	Journal of Environmental & Public Health	Peer	X	Qualitative Data Analysis	Low	No	Survey Primary Data Focus Groups Observations	Community Hygiene Education Social Behavioral Technical Health Capacity	Analysis Approach	Water Sanitation Hygiene	Urban	City	Access Behavior Change	Planning Implementation Evaluation	two urban slum communities in central Uganda; Kikulu zone in Kawempe division, Kampala district; and Kikooza zone in Mukono Municipality,	East Africa	2	Services Behavior Change	The three-year project, which was carried out between 2010 and 2013, had three phases: preparatory, implementation, and evaluation as described below		
EBSCO AP 2228	EBSCO	Why Do Water and Sanitation Systems for the Poor Still Fail? Policy Analysis in Economically Advanced Developing Countries.	M. Starkl, N. Brunner, T.A. Stenström	2013	Environmental Science & Technology	Peer		Case Study Statistics	High	Yes	Primary Data Secondary Data	Context Technical O&M Hygiene Community Social Financial Governance NGOs	Analysis	Water Sanitation	Rural Urban	Local	Sustainability	Case Study	60 case studies across; Mexico South Africa India	Central America South Africa South Asia	60				
EBSCO AP 30	EBSCO	The role of community participation for sustainable integrated neglected tropical diseases and water, sanitation and hygiene intervention programs: A pilot project in Tanzania.	S. Madon, M. Malecela, K. Mashoto, R. Donohue, G. Mubyazi, E. Michael	2018	ScienceDirect	Peer	X	Qualitative Data Analysis Statistics	High	Yes	Surveys Interviews Focus Groups	Socioeconomic Demographic Education Governance Health Technical	Approach Framework	WASH	Rural	Local	Health	Planning Case Study	Rufiji (as intervention district) and Mkuranga (used as a control) in the Coast (Pwani) Region of Tanzania	East Africa	1	Health			
EBSCO AP 324	EBSCO	The social benefits of water and sanitation projects in Northern Colombia: Cost-Benefit Analysis, the Water Poverty Index and beyond.	D. Azqueta, A. Montoya	2017	Wiley	Peer		Cost-Benefit Analysis Composite Scoring	Medium	No	Surveys Interviews Secondary data	Social Economic Environmental	Tool	Water Sanitation	Rural	Regional	Access	Case Study	Municipality of Moñitos,Department of Córdoba, in Northern Colombia	South America	1				
EBSCO AP 3313	EBSCO	A framework for planning of sustainable water and sanitation systems in peri-urban areas.	R. Tornqvist, A. Norstrom, E. Karrman, P.A. Malmqvist	2008	Water Science & Technology	Peer		Framework	Medium	No	Primary Data	Health Environmental Economic Technical Social Cultural NGOs Access	Approach	Water Sanitation		City	Sustainability	Planning							
EBSCO AP 41	EBSCO	Exploring a New Regionalism-Based Approach to Managing Drinking Water Systems in Rural Regions.	S.P. Breen	2018	Society & Natural Resources	Peer		Framework	Medium	Yes	Interviews Secondary Data	Governance Policy Environmental Economic Social	Approach	Water	Rural	Regional	Sustainability	Case Study	Kootenay, British Columbia, Canada	North America	1				
EBSCO AP 414	EBSCO	Designing a handwashing station for infrastructure-restricted communities in Bangladesh using the integrated behavioural model for water, sanitation and hygiene interventions (IBM-WASH)	K. Hulland, E. Leontsini, R. Dreibelbis, L. Unicomb, A. Afroz, N. Dutta, D. Nizame, S. Luby, P. Ram, R. Winch	2013	BMC Public Health	Peer	X	Behavioral Model Qualitative Coding	Medium	No	Interviews	Context Behavioral Technical	Framework Tool	Hygiene	Rural Urban	Local	Access	Planning Evaluation	Mohammadpur (Urban) and Kishoreganj (Rural), Bangladesh	South Asia	2				

EBSCO AP 428	EBSCO	Inclusive water poverty index: a holistic approach for helping local water and sanitation services planning.	J. Kini	2017	Water Policy	Peer		Composite Scoring	Medium	No	Secondary Data Interviews	Environmental Access Management Social Financial	Framework Approach	Water Sanitation	Rural	Regional	Access	Case Study	Niabouri, Silly and To municipalities in Burkina Faso	West Africa	3				

Document Information								Method						Scope				Application					Resources		
**Document ID**	**Database**	**Title**	**Author(s)**	**Date**	**Publication**	**Document Type**	**Open Access**	**Method Analysis**	**Analytical Complexity**	**Interaction Analysis**	**Data Source Analysis**	**Factors**	**Method Application**	**Aspects of WASH**	**Rural / Urban**	**Context**	**OutcomeScope**	**Project Application**	**Location**	**Location Region**	**Location Count**	**Study Impacts**	**Time**	**Human Resources**	**Cost**
EBSCO AP 508	EBSCO	A decision support model to aid the management of crises in urban water supply systems (the UWC-MODEL).	W. Da Silva, M. Souza	2017	Urgan Water Journal	Peer		Framework	High	Yes	Secondary Data	Demographic<br>Technical<br>Policy<br>Governance<br>Financial<br>Context<br>Climate<br>Socioeconomic	Framework	Water	Urban	Regional	Sustainability	Case Study	Administrative Region of Brasilia, Brazil	South America	1				
EBSCO AP 511	EBSCO	Harnessing the plurality of actor frames in social-ecological systems: ecological sanitation in Bolivia.	M. Eelderink, J. Vervoort, D. Snel, F.D. Castro	2017	Development in Practice	Peer	X	Statistics	High	No	Interviews<br>Primary Data<br>Observation	Health<br>Environmental<br>Economic<br>O&M<br>Social	Framework	Sanitation	Rural	Local	Access	Evaluation	San Pedro, Bolivia	South America	1				
EBSCO AP 774	EBSCO	Sustainable community-based drinking water systems in developing countries: stakeholder perspectives.	M. Aslam, M. Adil, S. Mirza, D.Frigon	2016	Journal of Water Supply: Research and Technology	Peer		AHP	High	No	Surveys	Technical<br>Environmental<br>Economic<br>Social<br>Institutional	Analysis	Water	Urban	Local	Coordination	Case Study	Peshawar, Pakistan	South Asia	1				
IRC 1070	IRC	Multiple-use water supply systems: do the claims stack up? : evidence from Bangladesh	M. Fontein, J. Webster, P. Trawick	2010	Waterlines	Peer		Framework	Low	No	Interviews<br>Observations<br>Secondary Data	Functionality<br>Community<br>Financial<br>Institutional<br>O&M<br>Management<br>Capacity	Framework	Water	Rural	Local	Access<br>Sustainability	Case Study<br>Evaluation	Bashubehar, Magbari and Chandaikona; Bogra, north-west Bangladesh.	South Asia	3	Services<br>Users			
IRC 1073	IRC	Between rural and urban: towards sustainable management of water supply systems in small towns in Africa	P. Moriarty, T. Patricot, T.F. Bastemeijer, J. Smet, C. Voorden	2002	IRC WASH	Org	X	Case Study	Medium	Yes	Secondary Data	Context<br>Technical<br>Community<br>Management<br>Governance<br>Institutional<br>Monitoring<br>Regulations<br>O&M<br>Financial<br>Private Sector<br>Political<br>Capacity<br>Policy<br>Donors<br>Environmental<br>Hygiene<br>Planning	Analysis	Water	Rural<br>Urban	City	Access<br>Sustainability	Case Study	Tanzania<br>Sudan<br>Ghana<br>Senegal<br>Benin	West Africa<br>East Africa<br>North Africa	5				
IRC 1075	IRC	Assessing the "Plus" of successful community-managed water supply programs in India	M. Snehalatha, S. Smits, S. Jasthi, P. Hutchings,R. Poonia, C. Daniel, C. Dash	2015	Australian AID: Department of Foreign Affairs and Trade (DFAT)	Grey	X	Qualitative Data Analysis	High	No	Secondary Data<br>Interviews<br>Focus Groups<br>Surveys	Management<br>Capacity<br>Coordination<br>Cultural<br>Economic<br>Donors<br>Financial<br>Governance<br>Technical<br>Implementation<br>Monitoring<br>Planning<br>Political<br>Private Sector<br>Services<br>Regulations	Analysis	Water	Rural	Local	Sustainability	Case Study	20 communities in India	South Asia	20				
IRC 1077	IRC	Norms and attitudes towards ecosan and other sanitation systems : desk study by a group of experts on ecological sanitation	J.-O. Drangert	2004	EcoSanRes Stockholm Environment Institute	Grey	X	Qualitative Data Analysis	High	No	Expert Opinion<br>Secondary Data<br>Observations<br>Interviews	Technical<br>Regulations<br>Economic<br>Health<br>Cultural<br>Social<br>Behavioral<br>Management<br>O&M<br>Networks	Analysis	Sanitation	Urban	City	Behavior Change	Case Study	Addis Ababa, Ethiopia<br>Cuernavaca, Mexico<br>Kisumu, Kenya<br>Dar es Salaam, Tanzania<br>Kabale, Uganda	East Africa<br>Central America	5				
IRC 1089	IRC	Assessing sustainability of community management of rural water systems in the developing world	R. Schweitzer, J. Mihelcic	2012	Journal of Water, Sanitation and Hygiene for Development	Peer		Composite Scoring<br>Statistics	High	No	Primary Data<br>Interviews	Community<br>Users<br>Governance<br>Financial<br>O&M<br>Functionality	Tool	Water	Rural	Local	Sustainability	Evaluation	Dominican Republic	Caribbean	1				
IRC 1122	IRC	Open planning of sanitation systems	R. Kvarnström, E. Petersens	2004	EcoSanRes Stockholm Environment Institute	Grey	X	Multi-Criteria Decision Analysis	Low	No	Expert Opinion<br>Secondary Data<br>Focus Groups	Networks<br>Hygiene<br>Environmental<br>Financial<br>Management<br>Technical<br>Social<br>Cultural<br>Institutional<br>O&M<br>Monitoring<br>Policy	Framework<br>Approach	Sanitation	Rural<br>Urban	Local	Sustainability	Planning	Vadsbro, Sweden	North Europe	1				
IRC 1201	IRC	Research into financial and institutional structures to support the functionality and sustainability of rural hill water systems	N.P. Kathmandu	2010	WaterAID	Org	X	Qualitative Data Analysis	Medium	No	Interviews<br>Secondary Data<br>Observations	O&M<br>Social<br>Environmental<br>Financial<br>Functionality<br>Capacity<br>Institutional<br>Monitoring<br>Community<br>Governance	Analysis	Water	Rural	Local	Sustainability	Evaluation	19 villages in 5 districts in Nepal Sankhuwa, Sabha, Dhading, Baglung, Kaski	South Asia	19				
IRC 122	IRC	Learning alliance approach: final report	J. Nkum	2014	IRC WASH	Org	X	Learning Alliances<br>Qualitative Data Analysis<br>Collective Action	Medium	No	Secondary Data<br>Interviews<br>Focus Groups	Capacity<br>Coordination<br>Financial<br>Governance<br>Implementation<br>Policy<br>Networks<br>NGOs<br>Participation<br>Policy<br>Political	Approach	Water	Rural	Regional<br>National	Coordination<br>Sustainability	Evaluation	Four pilot regions in Ghana; Northern, Brong Ahafo and Volta<br>Three pilot districts in Ghana; Gonja East in the Northern region; Sunyani West in the Brong Ahafo region; and Akatsi in the Volta region	West Africa	7	Coordination<br>Policy			
IRC 1266	IRC	Governance, sustainability and decision making in water and sanitation management systems	M.A. Iribarnegaray, L. Seghezzo	2012	Sustainability	Peer	X	Composite Scoring	Medium	No	Secondary Data<br>Interviews<br>Observations	Economic<br>Coordination<br>Regulations<br>Participation<br>Implementation<br>Capacity<br>Institutional<br>Behavioral<br>Social<br>Cultural<br>Governance<br>Networks	Tool	Water	Urban	City	Sustainability	Evaluation	Salta, Argentina	South America	1				

Document Information								Method						Scope				Application					Resources		
Document ID	Database	Title	Author(s)	Date	Publication	Document Type	Open Access	Method Analysis	Analytical Complexity	Interaction Analysis	Data Source Analysis	Factors	Method Application	Aspects of WASH	Rural / Urban	Context	Outcome Scope	Project Application	Location	Location Region	Location Count	Study Impacts	Time	Human Resources	Cost
IRC 133	IRC	Scaling up innovations through learning alliances : an introduction to the approach	S. Smits, P. Moriarty, C. Fonseca, T. Schouten	2007	IRC WASH	Org	X	Learning Alliances<br>Collective Action	Medium	Yes	Primary Data<br>Secondary Data<br>Expert Opinion	Capacity<br>Context<br>Coordination<br>Cultural<br>Financial<br>Governance<br>Implementation<br>Institutional<br>Monitoring<br>Networks<br>Participation<br>Planning<br>Policy<br>Political	Approach	WASH	Rural<br>Urban	Local<br>Regional<br>National<br>Sector	Coordination<br>Sustainability	Implementation	Cali, Columbia<br>Egypt<br>Palestine<br>Jordan<br>South Africa	South America<br>Middle East<br>South Africa	5	Uptake<br>Coordination<br>Policy	longer start-up period is needed: one year rather than the usual 3-6 months. This implies a need for advocacy to find funding for more enlightened approaches than conventional linear projects, and to take into account the quality of processes rather than purely quantitative outputs.	Team of 3 full time staff plus partner supported coordinators in two countries. Teams of 2 persons per region, dedicating about 25% of their time to process facilitation. 1 full time equivalent per country for process facilitation in addition to specialists and project management.	
IRC 152	IRC	Explaining the inter village variations in drinking water provision: factors influencing costs and service levels in rural Andhra Pradesh	R. Reddy	2012	IRC WASH	Org	X	Statistics	High	Yes	Secondary Data	Demographic<br>Social<br>Economic<br>Technical<br>Environmental<br>Climate<br>Institutional<br>Participation<br>Governance<br>Planning<br>Financial<br>Capacity	Analysis	Water	Rural	Local	Sustainability	Case Study	India<br>Ghana<br>Mozambique<br>Burkina Faso	South Asia<br>East Africa<br>West Africa	4				
IRC 153	IRC	ONEWASH Plus Sustainability Checks First Annual Report	M. Adank, E. Defere, J. Butterworth	2015	IRC WASH	Org	X	Composite Scoring	Medium	No	Primary Data<br>Surveys	Functionality<br>Services<br>Institutional<br>Financial<br>Environmental<br>Social<br>Infrastructure<br>Management<br>Capacity<br>Monitoring<br>O&M<br>Policy<br>Participation<br>Economic<br>Hygiene	Tool	Water<br>Sanitation<br>WASH	Urban<br>Rural	Local	Sustainability	Evaluation<br>Planning	Project Towns in Ethiopia<br>Maksegnit<br>Abomsa<br>Sheno<br>Welenchiti<br>Adishihu<br>Wukro	East Africa	6				
IRC 175	IRC	Making cities open defecation free : systematic approach in Maharashtra	Department, Maharashtra (State) Urban Development; University, CEPT; Self-Government, All India Institute of Local	2016	Government of Maharashtra, India	Grey	X	Framework	Low	No	Surveys<br>Observations<br>Focus Groups	Institutional<br>Access<br>Behavioral<br>Capacity<br>Community<br>Context<br>Coordination<br>Education<br>Financial<br>Health<br>Hygiene<br>Implementation<br>Management<br>NGOs<br>O&M<br>Participation<br>Planning<br>Political<br>Services<br>Social	Approach	Sanitation<br>Hygiene	Urban	Local<br>City	Behavior Change<br>Access<br>Sustainability	Planning<br>Implementation	19 cities, 51 councils and 1 corporation in Maharashtra District, India	South Asia	1	Behavior Change<br>Uptake<br>Services<br>Financial<br>Policy			
IRC 253	IRC	Multiple-use services as an alternative to rural water supply services : a characterisation of the approach	S. Smits, B. van Koppen, P. Moriarty, J. Butterworth	2010	Water Alternatives	Peer	X	Framework<br>Case Study	Medium	No	Surveys<br>Interviews<br>Observations<br>Focus Groups	Technical<br>Institutional<br>Financial<br>Environmental	Analysis	Water	Rural	Local	Access<br>Sustainability	Case Study	30 villages across 8 countries;<br>Bolivia<br>Colombia<br>Ethiopia<br>India<br>Nepal<br>South Africa<br>Thailand<br>Zimbabwe	South America<br>East Africa<br>South Africa<br>South Asia<br>Southeast Asia	8				
IRC 278	IRC	A livelihood approach to water interventions in rural areas and implications for Multiple Use Systems	J.M. Faurès, G. Santini, A.N. Villemarceau	2008	International Symposium of Multiple-Use Water Services	Peer	X	Framework	Low	No	Secondary Data	Access<br>Agriculture<br>Context<br>Economic<br>Climate<br>Technical	Framework	Water	Rural	Local	Access	Framework	Sub-Saharan Africa	East Africa<br>West Africa<br>Middle Africa<br>South Africa	4				
IRC 330	IRC	A service delivery approach for rural water supply in Timor-Leste : institutional options and strategy	J. Willetts	2012	University of Technology Sydney	Grey	X	Framework<br>Building Blocks<br>Qualitative Data Analysis	High	No	Secondary Data<br>Interviews	Community<br>Services<br>Capacity<br>Monitoring<br>Policy<br>Technical<br>Regulations<br>Education<br>Infrastructure<br>Financial<br>Governance	Framework	Water	Rural	Local	Sustainability	Framework	Timor-Leste	Southeast Asia	1				

IRC 348	IRC	Adopting a sustainable livelihoods approach to water projects : implications for policy and practice	A. Nicol	2000	Overseas Development Institute	Grey	X	Sustainable Livelihoods Framework	Medium	Yes	Expert Opinion	Access<br>Agriculture<br>Capacity<br>Climate<br>Community<br>Context<br>Coordination<br>Demographic<br>Donors<br>Economic<br>Education<br>Environmental<br>Financial<br>Gender<br>Governance<br>Institutional<br>Management<br>NGOs<br>Participation<br>Policy<br>Political<br>Private Sector<br>Regulations<br>Services	Framework	Water	Rural<br>Urban	Local	Access	Implementation							

Document Information								Method						Scope				Application					Resources		
Document ID	Database	Title	Author(s)	Date	Publication	Document Type	Open Access	Method Analysis	Analytical Complexity	Interaction Analysis	Data Source Analysis	Factors	Method Application	Aspects of WASH	Rural / Urban	Context	OutcomeScope	Project Application	Location	Location Region	Location Count	Study Impacts	Time	Human Resources	Cost
IRC 356	IRC	A life-cycle approach to improve the sustainability of rural water systems in resource-limited countries	S.A. Jones, A. Anya, N. Stace, L. Weir	2012	Challenges	Peer	X	Lifecycle Analysis<br>Framework<br>Decision Trees	Medium	No	Secondary Data	Technical<br>Environmental<br>Institutional<br>Community<br>Management<br>Users<br>Financial<br>Health	Framework Approach	Water	Rural	Local	Sustainability	Planning	Jhumka, Indrapur, and Panchakanya in Nepal	South Asia	3				
IRC 362	IRC	Integrate at the top, involve at the bottom – the household-centred approach to environmental sanitation	C. Lüthi, A. Morel, E. Tilley	2008	IRC WASH	Peer	X	Framework	Medium	No	Expert Opinion Workshop	Technical<br>Users<br>Social<br>Financial<br>Institutional<br>Access<br>Governance	Approach	Sanitation	Urban	Local	Access	Evaluation	Costa Rica<br>Burkina Faso<br>Kenya<br>Tanzania<br>Laos<br>Nepal	Central America<br>West Africa<br>East Africa<br>South Asia	6	Uptake	A one-day interactive community workshop ensures that the communities' views and knowledge is recognised and forms a valuable input to the assessment report that follows in the third planning step.		
IRC 364	IRC	The effectiveness and sustainability of two demand-driven sanitation and hygiene approaches in Zimbabwe	L. Whaley, J. Webster.	2011	Journal of Water, Sanitation and Hygiene for Development	Peer		Case Study<br>Qualitative Data Analysis	Medium	No	Surveys<br>Interviews<br>Focus Groups	Demographic<br>Economic<br>Cultural<br>Social<br>Community<br>Health<br>Hygiene<br>Financial<br>Climate<br>Infrastructure<br>NGOs	Analysis	Sanitation<br>Hygiene	Rural	Local	Behavior Change<br>Sustainability	Evaluation	The study was carried out in three districts of Zimbabwe:Chiredzi district, Chipinge district and Mutoko district	East Africa	3	Behavior Change			
IRC 372	IRC	A life-cycle capacity-based approach to allocating investments in municipal sanitation infrastructure	G.E. Louis, L. Magpili	2007	Structure and Infrastructure Engineering	Peer		Composite Scoring	High	No	Secondary Data	Services<br>Institutional<br>Capacity<br>Technical<br>Economic<br>Financial<br>Energy<br>Environmental<br>Social<br>Cultural	Tool	Sanitation	Urban	Local	Sustainability	Case Study	Manila, Philippines<br>Mombasa, Kenya	East Africa<br>Southeast Asia	2				
IRC 373	IRC	From beneficiaries to businesses to the big picture: Monitoring for sustainability in market-based approaches to sanitation	D. Sparkman	2013	IRC, Water For People	Grey	X	Composite scoring	Low	No	Interviews<br>Observations<br>Surveys	Access<br>Services<br>Private Sector<br>Community<br>NGOs<br>Infrastructure<br>Financial<br>Regulations<br>Political	Tool	Sanitation	Rural<br>Urban	Local<br>Regional	Sustainability	Case Study	Blantyre, Malawi	East Africa	1				
IRC 478	IRC	Flores revisited : sustainability, hygiene and use of community-managed water supply and sanitation and the relationships with project approaches an...	C. Sijbesma, K. Sari, N. Shatifan, R. Walujan, I. Mukherjee, R. Hopkins	2002	WSP-EAP, IRC WASH	Grey	X	Method for Participatory Assessment (MPA)<br>Statistics	High	Yes	Focus Groups<br>Workshops<br>Primary Data<br>Secondary Data<br>Observations	Social<br>Economic<br>Health<br>Technical<br>Environmental<br>Community<br>Financial<br>Management<br>Access<br>Hygiene<br>Behavioral<br>Institutional<br>Participation<br>Gender	Analysis	Water<br>Sanitation<br>Hygiene	Rural	Local	Sustainability	Evaluation	63 Communities in Flores, NTT, Indonesia	Oceania	1				
IRC 539	IRC	Sanitation 21 : simple approaches to complex sanitation : a draft framework for analysis	J. Parkinson, C. Lüthi, D. Walther	2006	The International Water Association (IWA)<br>The Swiss Federal Institute of Aquatic Science and Technology (Eawag)<br>The Deutsche Gesellschaft für Internationale Zusammenarbeit (GIZ)<br>Sustainable Sanitation Alliance	Grey	X	Framework	Low	No	Secondary Data<br>Primary Data<br>Surveys<br>Focus Groups	Planning<br>NGOs<br>Networks<br>Institutional<br>Private Sector<br>Policy<br>Regulations<br>O&M<br>Infrastructure<br>Technical<br>Financial<br>Services<br>Social<br>Monitoring<br>Implementation<br>Capacity	Framework	Sanitation	Urban	City	Sustainability	Planning							
IRC 575	IRC	External factors influencing the sustainability of micro and small piped water enterprises in Kenya's peri-urban areas	E. Mukulu, L. Oyugi, S. Mwarania	2011	International Journal of Humanities and Social Science	Peer	X	Qualitative data analysis<br>Statistics	High	No	Surveys<br>Interviews<br>Observations	Social<br>Political<br>Regulations<br>Economic<br>Private Sector	Analysis	Water	Urban	City	Sustainability	Case Study	Nairobi, Mombasa and Kisumu, Kenya	East Africa	3				
IRC 601	IRC	Factors influencing knowledge and practice of hygiene in Water, Sanitation and Hygiene (WASH) programme areas of Bangladesh Rural Advancement Committee	T. Akter, A. Mehrab	2014	Rural and Remote Health	Peer	X	Qualitative data analysis	Medium	No	Interviews<br>Observation	Hygiene<br>Users<br>Behavioral<br>Financial<br>Environmental<br>Social<br>Education<br>Participation<br>Demographic<br>Services	Analysis	Sanitation<br>Hygiene	Rural	Local	Behavior Change	Case Study	12 Villages across six upazilas in BRAC programs in Bangladesh	South Asia	6				
IRC 63	IRC	Promoting harmonized monitoring for the WASH sector : the Rural Water and Sanitation Information System SIASAR as an example	A. Rodriguez Serrano	2018	IRC WASH, World Bank	Grey	X	SIASAR<br>Composite Scoring	Medium	No	Survey	Infrastructure<br>Technical<br>Financial<br>O&M<br>Services<br>Access<br>Demographic<br>Health<br>Hygiene<br>Capacity	Tool	Water	Rural	Local	Sustainability	Evaluation	Mexico<br>Dominican Republic<br>Costa Rica<br>Nicaragua<br>Panama<br>Peru<br>Brazil	Central America<br>South America<br>Caribbean	3	Uptake			

IRC 636	IRC	Sustainable replication and scaling up of small piped water networks : technical assistance consultant's report	University of Tokyo Civil Engineering -Tokyo, JP, UTCE	2008	Asian Development Bank	Grey	X	Case Study Qualitative Data Analysis	High	No	Interviews Observations Secondary Data	Context NGOs Institutional Capacity Financial Regulations Monitoring O&M Management Private Sector Social Services	Case Study	Water	Urban	City	Sustainability	Case Study	Four utilities in Manila, Philippines (2); Ahmedabad, India; Tien Giang, Vietnam	South Asia Southeast Asia	4				

Document Information								Method						Scope				Application					Resources		
Document ID	Database	Title	Author(s)	Date	Publication	Document Type	Open Access	Method Analysis	Analytical Complexity	Interaction Analysis	Data Source Analysis	Factors	Method Application	Aspects of WASH	Rural / Urban	Context	Outcome Scope	Project Application	Location	Location Region	Location Count	Study Impacts	Time	Human Resources	Cost
IRC 637	IRC	Factors influencing water system functionality in Nigeria and Tanzania : a regression and Bayesian network analysis	R. Cronk, J. Bartram	2017	Environmental Science & Technology	Peer		Statistics	High	Yes	Secondary Data	Environmental<br>Financial<br>Functionality<br>Agriculture<br>Management<br>Technical<br>Context	Analysis	Water	Rural<br>Urban	Local<br>City	Sustainability	Evaluation	82,503 systems in Tanzania and Nigeria	East Africa<br>West Africa	2				
IRC 70	IRC	Organizing framework for functional national WASH monitoring and evaluation systems	UNICEF; IRC; Akvo	2016	UNICEF, IRC WASH, Akvo	Grey	X	Framework	Low	No	-	Institutional<br>Capacity<br>Coordination<br>Financial<br>Implementation<br>Monitoring	Framework<br>Tool	WASH	Rural<br>Urban	National	Sustainability	Evaluation							
IRC 879	IRC	Sustainable community water : managing supply systems in the mid-hills of Nepal	B.S. Bhandari	2005	Water Policy	Peer		Qualitative data analysis<br>Statistics<br>Composite Scoring	High	No	Surveys<br>Observation<br>Focus Groups	NGOs<br>Users<br>Management<br>Participation<br>Community<br>Access<br>Services<br>Gender	Analysis	Water	Rural	Local	Sustainability	Evaluation	Baglung and Kavre, Nepal	South Asia	1				
IRC 912	IRC	Looking back to move forward in strength : monitoring of water system sustainability	K. Fogelberg	2009	Knowledge Management for Development Journal	Peer	X	Composite Scoring	Low	No	Interviews<br>Observation<br>Primary Data	Access<br>Management<br>Financial<br>O&M<br>Users<br>Hygiene<br>Environmental	Tool	Water	Rural<br>Urban	Regional	Sustainability	Evaluation	Honduras<br>Guatemala<br>Bolivia<br>India<br>Malawi	Central America<br>South America<br>West Africa<br>South Asia	3	Uptake	The monitoring exercise is scheduled during the dry season in all countries, as we are most interested in seeing if water systems are able to meet year rounds, needs and thus, visit when they are under the most stress. A typical two-week exercise is structured as follows:	The structure of the groups – an external volunteer, local partner, Water For People staff, and university students (when available) is created precisely to encourage this sharing of information, technical expertise, and creative thinking. The last day of the exercise is typically a presentation	Finance: The majority of costs are borne by external participants, the World Water Corps volunteers
IRC ASG15	IRC	Strengthening the sanitation market system : WaterSHED's Hands-Off experience	M.W. Jenkins, L. McLennan, G. Revell, A. Salinger	2019	IRC WASH: All Systems Go Conference Preceedings	Peer	X	Market-Based approach	Low	No	Primary Data<br>Secondary Data	Policy<br>Technical<br>Private Sector<br>Financial<br>Gender<br>Capacity<br>Governance<br>Coordination<br>NGOs<br>Donors<br>Regulations	Approach	Sanitation	Rural	Local<br>Regional<br>National	Access	Implementation	Cambodia	Southeast Asia	1	Access	1. Start-up – design and piloting [2009-2011] 2. Scale-up, maturation, replication & consolidation [2012-2017] 3. Sustainability [2018-2020]		
IRC ASG2	IRC	Facilitating local strengthening of WASH systems : whose understanding counts?	M.E. Mussa, T.N. Michael, B.G. Hailegiorgis, M.J. Morris, J.A. Butterworth, L. Henry	2019	IRC WASH: All Systems Go Conference Preceedings	Peer	X	Learning Alliances	Low	No	Primary Data	Coordination<br>Monitoring<br>Infrastructure<br>Financial<br>O&M<br>Management	Approach<br>Framework	Water<br>Sanitation	Rural<br>Urban	Local<br>Regional	Sustainability	Planning<br>Implementation	Four locations in Ethiopia:<br>Mile, Afar Woreda<br>South Ari SNPPR Woreda<br>Woliso<br>Debre Birhan	East Africa	4	Uptake	Two dedicated week-long capacity-building workshops for the facilitators were held in September 2017 and April 2018, with further capacity-building and support arranged subsequently		
IRC ASG20	IRC	Kick-starting WASH systems in newly established local authorities : an experience from Nepal	W. Tillett, P. Bastola, S. Gautam	2019	IRC WASH: All Systems Go Conference Preceedings	Grey	X	Systems Approach<br>Composite Scoring	Medium	No	Surveys<br>Primary Data<br>Secondary Data<br>Workshops	Institutional<br>Technical<br>Capacity<br>Environmental<br>Financial<br>Services<br>Planning<br>Implementation<br>Social<br>Institutional<br>Monitoring	Approach	WASH	Rural	Regional	Sustainability	Case Study	Chitwan District, Nepal	South Asia	1	Policy	At the time of writing this paper, the SSI 'upstream' component of the project in Nepal had only been running for 10 months, with a strong focus to date on assessment phase activities.		
IRC ASG22	IRC	Developing consumer markets within rural WASH systems	A. Smith, J. Butterworth	2019	IRC WASH: All Systems Go Conference Preceedings	Peer	X	Market-Based approach<br>Case Study	Medium	No	Secondary Data	Private Sector<br>Economic<br>Government<br>Hygiene<br>NGOs<br>Policy<br>Networks	Analysis	Water<br>Sanitation	Rural	Local<br>Regional<br>National<br>Sector	Sustainability	Evaluation							
IRC ASG24	IRC	Fostering WASH system changes in Haiti using principles from the market systems sector	A. Montangero, V. Cavin, M. Pierre, L. Blaser	2019	IRC WASH: All Systems Go Conference Preceedings	Peer	X	Market-Based approach	Medium	No	Focus Groups<br>Expert Opinion	Private Sector<br>Governance<br>Users<br>Management<br>Planning<br>O&M<br>Monitoring<br>Coordination<br>Financial<br>Networks<br>Institutional<br>Policy<br>Regulations	Approach	WASH	Rural<br>Urban	National<br>Regional	Sustainability	Planning	Haiti	Caribbean	1	Policy<br>Coordination			

IRC ASG26	IRC	The Sanitation Cityscape Conceptual Framework : understanding urban sanitation systems	P. Scott	2019	IRC WASH: All Systems Go Conference Preceedings	Peer	X	Framework	Low	Yes	Primary Data	Institutional<br>Policy<br>Capacity<br>Financial<br>Planning<br>Monitoring<br>Services<br>Infrastructure	Framework	Sanitation	Urban	City	Sustainability	Implementation	One small town in Ethiopia	East Africa	1				

Document Information								Method						Scope				Application					Resources		
Document ID	Database	Title	Author(s)	Date	Publication	Document Type	Open Access	Method Analysis	Analytical Complexity	Interaction Analysis	Data Source Analysis	Factors	Method Application	Aspects of WASH	Rural / Urban	Context	Outcome Scope	Project Application	Location	Location Region	Location Count	Study Impacts	Time	Human Resources	Cost
IRC ASG28	IRC	Keeping community managed handpump systems going	R. Carter	2019	IRC WASH: All Systems Go Conference Preceedings	Peer	X	Framework	Low	No	Expert Opinion<br>Observations	Community<br>Technical<br>Financial<br>Context<br>Regulations<br>Capacity<br>Management<br>Institutional	Framework	Water	Rural	Local	Sustainability	Evaluation							
IRC ASG3	IRC	Strengthening water quality monitoring systems in Asutifi North, Ghana	A. Karon, C. Delaire, C. Mcleod, R. Peletz, R. Khush	2019	IRC WASH: All Systems Go Conference Preceedings	Peer	X	Institutional Mapping<br>Systems Approach<br>Qualitative Data Analysis	High	No	Primary Data<br>Secondary Data<br>Interviews<br>Surveys	Economic<br>Functionality<br>Health<br>Institutional<br>Financial<br>Networks<br>Technical<br>O&M<br>Environmental<br>Governance<br>Capacity<br>Political<br>Monitoring	Approach	Water	Rural	Regional	Sustainability	Evaluation	Asutifi North, Ghana	West Africa	1	Uptake			
IRC ASG5	IRC	Developing district WASH investment plans, a cornerstone of the district wide approach	P. Kamuyumbu, M. Kwizera	2019	IRC WASH: All Systems Go Conference Preceedings	Peer	X	District-Wide Approach<br>Systems Approach	Medium	No	Primary Data<br>Secondary Data	Infrastructure<br>Services<br>Capacity<br>Environmental<br>O&M<br>Monitoring<br>Political	Approach	WASH	Rural	Regional	Sustainability	Planning	Rulindo District, Rwanda	West Africa	1	Policy<br>Services	Six years (project duration)		
IRC ASG7	IRC	Positioning WASH services within a systems framework and demonstrating achievement of open defecation free status at scale : an example from the Indian State of Bihar	A. AUmar, B. Satviki Varma	2019	IRC WASH: All Systems Go Conference Preceedings	Grey	X	Systems Approach	Low	No	Workshops	Access<br>Behavioral<br>Capacity<br>Community<br>Education<br>Financial<br>Gender<br>Governance<br>Technical<br>Hygiene<br>Implementation<br>Institutional<br>Monitoring<br>Participation<br>Political<br>Private Sector<br>Social	Approach	Sanitation	Rural	Local	Access	Approach	Pusa block of Samastipur district, Bihar, India	South Asia	1	Services			
IRC ASG8	IRC	Performance-based financing and capacity building to strengthen WASH systems in Mozambique : early findings	L. Rudge	2019	IRC WASH: All Systems Go Conference Preceedings	Grey	X	Building Blocks<br>Qualitative Data Analysis<br>Mapping	Medium	No	Interviews<br>Surveys<br>Workshops	Institutional<br>Services<br>Infrastructure<br>Monitoring<br>Planning<br>Financial<br>Regulations<br>Capacity<br>Coordination	Approach<br>Analysis	WASH	Rural	Regional	Sustainability	Planning<br>Implementation<br>Evaluation	20 districts in two provinces of Mozambique (Nampula and Zambezia)	East Africa	2	Uptake<br>Services			
IRC ASG9	IRC	Digging deep behind the complexities of sustainable water supply in Nepal	A. Adhikari	2019	IRC WASH: All Systems Go Conference Preceedings	Grey	X	Qualitative Data Analysis<br>Financial Analysis	Medium	No	Primary Data<br>Interviews<br>Focus Groups	Technical<br>Financial<br>Governance	Analysis	Water	Rural	Regional	Sustainability	Evaluation	Sarlahi and Rautahat districts in Terai and Nuwakot, and Gorkha and Dhading districts in the Hills region (Nepal)	South Asia	5				
OECD 6	OECD	Enhancement of domestic financial support mechanisms for water supply and sanitation in Moldova: Scenario analysis	OECD (Uncredited)	2017	OCED	Grey	X	Scenario Analysis	Low	No	Expert opinion	Social<br>Economic<br>Institutional<br>Financial	Analysis	Water<br>Sanitation	Rural<br>Urban	National	Sustainability	Planning	Moldova	East Europe	1				
SciElo 120	SciElo	Local and social management capacities for domestic water supply in rural communities of the Zamora Valley, Michoacán, Mexico	J.L. Pimentel-Equihua, M.A. Velázquez-Machuca, J. Palerm-Viqueira	2012	Agricultura, sociedad y desarrollo (Agriculture, Society and Development)	Peer	X	Framework	Medium	No	Interviews<br>Observation<br>Primary Data<br>Secondary Data	Management<br>Governance<br>Capacity<br>Financial<br>Monitoring	Analysis	Water	Rural	Local	Sustainability	Case Study	Six rural communities in the municipality of Zamora, in the northwest of Michoacá, Mexico	Central America	6				
SciElo 147	SciElo	Proposition of a system of performance indicators to evaluate the quality of sewage services	T.L. von Sperling, N. von Sperling	2013	Sanitary and Environmental Engineering	Peer	X	Delphi Panel<br>Framework	High	No	Secondary Data<br>Expert Opinions	Management<br>Economic<br>Financial<br>Environmental<br>Capacity<br>Infrastructure	Framework	Sanitation		National	Sustainability	Evaluation	Brazil	South America	1				
SciElo 221	SciElo	Water service delivery challenges in a small South African municipality: Identifying and exploring key elements and relationships in a complex social-ecological system	M.J.T. Weaver	2017	Water SA	Peer	X	Complex Social-Ecological Systems Approach<br>Causal Loop Diagramming	Medium	Yes	Expert Opinion<br>Surveys	Infrastructure<br>Technical<br>Governance<br>Users<br>Policy<br>NGOs<br>Social<br>Economic<br>Political<br>Financial<br>O&M<br>Capacity<br>Management	Approach	Water	Urban	Regional	Sustainability	Case Study	Makana Local Municipality, South Africa	South Africa	1	Uptake			
WEDC 2	WEDC	Soft systems methodology for performance measurement in the Uganda water sector	S. Kayaga	2008	IWA	Peer	X	Soft Systems Methodology<br>CATOWE	Low	No	Workshops	Networks<br>Coordination<br>Financial<br>Services<br>Political<br>Economic<br>Private Sector<br>NGOs<br>Donors	Analysis<br>Framework	Water<br>Sanitation		National	Sustainability	Evaluation	Uganda	East Africa	1	Uptake	3 days		
WEDC 7	WEDC	Livelihood factors, explaining water consumption in a (de facto) multiple uses water system in Colombia	I. Dominguez, W. Torres-Lopez, I. Restrepo-Tarquino, R. Oviedo-Ocana, I. Smout	2014	Loughborough University	Peer	X	Statistics	High	No	Primary data<br>Surveys	Agriculture<br>Socioeconomic<br>Technical	Tool	Water	Rural	Local	Access	Case Study	Colombia	South America	1				

World Bank 1	World Bank	Sustainability Assessment of Rural Water Service Delivery Models Findings of a Multi-Country Review	S. Smets, H. Lockwood, S. Mansour, S. Smits	2017	World Bank Group	Grey	X	Composite Scoring	Medium	No	Interviews<br>Secondary Data<br>Expert Opinion	Institutional<br>Financial<br>Management<br>Environmental<br>Monitoring<br>Regulations	Framework	Water	Rural	Regional-National	Sustainability	Case Study	Bangladesh<br>Benin<br>Brazil<br>China<br>Ethiopia<br>Ghana<br>Haiti<br>India<br>Indonesia<br>the Kyrgyz Republic<br>Morocco<br>Nepal<br>Nicaragua<br>the Philippines<br>Tanzania<br>Vietnam	South Asia<br>Central America<br>South America<br>Caribbean<br>East Africa<br>West Africa<br>Central Asia<br>East Asia<br>Southeast Asia	16				

Document Information								Method						Scope				Application					Resources		
Document ID	Database	Title	Author(s)	Date	Publication	Document Type	Open Access	Method Analysis	Analytical Complexity	Interaction Analysis	Data Source Analysis	Factors	Method Application	Aspects of WASH	Rural / Urban	Context	Outcome Scope	Project Application	Location	Location Region	Location Count	Study Impacts	Time	Human Resources	Cost
World Bank 218	World Bank	Introducing FOAM	Y. Coombes, J. Devine	2010	The World Bank	Grey	X	Framework	Medium	No	Secondary Data	Technical<br>Financial<br>Behavioral<br>Regulations<br>Social	Approach<br>Framework	Hygiene	Rural<br>Urban	Local<br>City	Sustainability	Planning<br>Implementation	China (Sichuan)<br>China (Shaanxi)<br>Ghana<br>India (Kerala)<br>Kyrgyzstan<br>Madagascar<br>Peru<br>Senegal<br>Tanzania<br>Uganda<br>Vietnam	East Asia<br>West Africa<br>South America<br>Central Asia<br>East Africa<br>Southeast Asia	3				
World Bank 355	World Bank	Community Slum Sanitation in India	World Bank Group	2016	The World Bank	Grey	X	Enabling Environment Framework	Low	No	Surveys<br>Primary Data<br>Secondary Data	Political<br>Institutional<br>Infrastructure<br>Community<br>Financial<br>Regulations<br>Health<br>Participation<br>Gender<br>Coordination<br>Context<br>Management<br>Monitoring	Approach	Sanitation	Urban	City<br>Local	Access<br>Sustainability	Planning<br>Implementation<br>Evaluation	Six cities in India: Ahmedabad, Gujarat; Bhopal Madhya, Pradesh; Kalyani, West Bengal; Mumbai, Maharashtr; Pune, Maharashtra; Tiruchirappalli (or Trichy), Tamil Nadu	South Asia	6				

												NGOs<br>Capacity													
World Bank<br>408	World Bank	Expanding Access to<br>Improved Sanitation for<br>the Poor	S. Saruta, A. Geaneotes, R. Gilles,<br>R. Muana, F. Mills, T. Masuoka	2018	The World Bank /<br>IFC	Grey	X	Qualitative Data<br>Analysis	Medium	No	Surveys<br>Focus Groups<br>Interviews	Technical<br>Institutional<br>Services<br>Private Sector<br>Financial<br>NGOs<br>Governance<br>Economic<br>Demographic<br>Social	Analysis<br>Framework	Sanitation	Urban	City	Access	Case Study	Four districts in<br>Philippines around<br>metro Manila<br>(Pampanga, Rizal,<br>Laguna, Manila)	Oceania	4				
World Bank<br>587	World Bank	Papua New Guinea<br>Rural WaSH<br>Sustainability Study	K. Clark, N. Francis, G. Pigolo, M.<br>Bond, P. Tyndale-Biscoe	2017	The World Bank	Grey	X	Qualitative Data<br>Analysis<br>Composite Scoring	Medium	No	Survey<br>Interviews<br>Observation<br>Focus Groups	Functionality<br>Management<br>O&M<br>Users<br>Financial<br>Community<br>Hygiene<br>Gender<br>Monitoring<br>Governance<br>Policy	Analysis	Water<br>Sanitation<br>Hygiene	Rural	Local	Sustainability	Evaluation	20 communities<br>across 4 provinces<br>(Eastern Highlands,<br>Central, Morobe,<br>Madang) in Papua<br>New Guinea	Oceania	4				
												Community<br>Private Sector													

WSP 1	WSP	Marching together with a Citywide Sanitation Stratergy	N.T. Utomo, M. Listyasari	2010	WSP	Grey	X	Framework	Medium	No	Workshops<br>Surveys<br>Secondary Data	NGOs<br>Policy<br>Capacity<br>Regulations<br>Financial<br>Monitoring	Approach	Sanitation	Urban	City	Access<br>Sustainability	Planning							

SI 4: RESULTS SUMMARY

Table S4-1: Results Summary Table - Methodology

Methodology Descriptors	Criteria					
	Method	Peer	Grey	Org	**Total**	%
Method[2]	Framework	21	12	8	**41**	**31%**
	Composite Scoring	12	10	8	**30**	**23%**
	Qualitative Data Analysis	11	10	2	**23**	**17%**
	Statistics	11	1	2	**14**	**11%**
	Social Network Analysis	8	2		**10**	**8%**
	Case Study	5	1	1	**7**	**5%**
	Systems Approach	3	2	2	**7**	**5%**
	AHP	6			**6**	**5%**
	Multi-Criteria Decision Analysis	5	1		**6**	**5%**
	System Dynamics	6			**6**	**5%**
	Market-Based Approach	5			**5**	**4%**
	Causal Loop Diagramming	4	2		**6**	**5%**
	Collective Action / Impact	1		3	**4**	**3%**
	Method for Participatory Assessment	2	1	1	**4**	**3%**
	Theory of Change		1	3	**4**	**3%**
	Building Blocks		2	1	**3**	**2%**
	Learning Alliances	1		2	**3**	**2%**
	Participatory Systems Mapping	2	1		**3**	**2%**
	Service Delivery Approach			3	**3**	**2%**
	SIASAR		3		**3**	**2%**
	Agent Based Modeling	2			**2**	**2%**
	Bayesian Networks	2			**2**	**2%**
	Financial Analysis		1	1	**2**	**2%**
	Lifecycle Analysis	2			**2**	**2%**
	Qualitative Comparative Analysis	2			**2**	**2%**
	Behavioral Model	1			**1**	**1%**
	CATOWE	1			**1**	**1%**
	Cost-Benefit Analysis	1			**1**	**1%**
	Decision Trees	1			**1**	**1%**
	Delphi Panel	1			**1**	**1%**
	District-Wide Approach / Systems Approach	1			**1**	**1%**
	Enabling Environment		1		**1**	**1%**
	GIS	1			**1**	**1%**
	Graphic Modeling	1			**1**	**1%**
	Institutional Mapping	1			**1**	**1%**

Mathematical optimization	1			**1**	**1%**
Remote sensing	1			**1**	**1%**
Scenario Analysis		1		**1**	**1%**
Shit Flow Diagrams			1	**1**	**1%**
Soft Systems Methodology	1			**1**	**1%**
Sustainable Livelihoods Framework		1		**1**	**1%**

Analytical Complexity[1]

	Peer	Grey	Org	**Total**	%
Low	13	10	10	**33**	**25%**
Medium	23	16	10	**49**	**37%**
High	39	9	3	**51**	**38%**

Interactions[1]

Peer	Grey	Org	**Total**	%
26	5	4	**35**	**26%**

Sources of data[2]

Data Source	Peer	Grey	Org	**Total**	%
Secondary Data	34	18	19	**71**	**53%**
Interviews	37	15	11	**63**	**47%**
Primary Data	20	7	10	**37**	**28%**
Observation	22	10	3	**35**	**26%**
Surveys	16	11	5	**32**	**24%**
Focus Groups	14	10	7	**31**	**23%**
Expert Opinion	13	10	5	**28**	**21%**
Workshops	1	5	0	**6**	**5%**

Factors[2]

Factor Name	Peer	Grey	Org	**Total**	%
Financial	46	31	21	**98**	**74%**
Technical	45	17	9	**71**	**53%**
Institutional	21	19	17	**57**	**43%**
Social	30	15	9	**54**	**41%**
Environmental	32	8	13	**53**	**40%**
Capacity	17	18	9	**44**	**33%**
Economic	30	6	4	**40**	**30%**
O&M	20	13	7	**40**	**30%**
Management	18	14	7	**39**	**29%**
Governance	20	9	7	**36**	**27%**
Community	21	10	3	**34**	**26%**

	Monitoring	7	16	11	**34**	**26%**
	Coordination	13	11	6	**30**	**23%**
	Services	10	15	3	**28**	**21%**
	Policy	10	7	10	**27**	**20%**
	Regulations	6	15	4	**25**	**19%**
	NGOs	11	10	1	**22**	**17%**
	Access	10	8	2	**20**	**15%**
	Infrastructure	9	6	5	**20**	**15%**
	Private Sector	8	9	3	**20**	**15%**
	Hygiene	7	9	3	**19**	**14%**
	Political	6	7	5	**18**	**14%**
	Functionality	12	3	2	**17**	**13%**
	Health	9	6	2	**17**	**13%**
	Networks	11	4	2	**17**	**13%**
	User	14	2	1	**17**	**13%**
	Planning	4	5	7	**16**	**12%**
	Context	9	4	2	**15**	**11%**
	Implementation	3	7	4	**14**	**11%**
	Behavioral	7	6	0	**13**	**10%**
	Education	6	5	2	**13**	**10%**
	Participation	3	5	5	**13**	**10%**
	Cultural	7	3	1	**11**	**8%**
	Demographic	4	3	1	**8**	**6%**
	Gender	2	5	1	**8**	**6%**
	Donors	3	2	2	**7**	**5%**
	Socioeconomic	7	0	0	**7**	**5%**
	Climate	4	1	1	**6**	**5%**
	Agriculture	3	1	1	**5**	**4%**
	Energy	5	0	0	**5**	**4%**
	Factor Count	Peer	Grey	Org	**Total**	%
	2	1	0	0	**1**	**1%**
	3	5	1	1	**7**	**5%**
	4	4	2	0	**6**	**5%**
	5	14	4	5	**23**	**17%**
	6	16	3	1	**20**	**15%**
	7	9	2	4	**15**	**11%**
	8	11	3	4	**18**	**14%**
	9	5	3	0	**8**	**6%**
	10	3	5	2	**10**	**8%**

Unless specified otherwise, each table represents counts of studies and % of total count of studies, n=133

Notes: 1: a-priori, deductive criteria; 2: open or emergent criteria

Note: Many descriptors represent non-exclusive criteria (i.e. studies can be classified under multiple attributes, % do not always sum to 100%)

Table SI 4-2: Results Summary Table - Scope

Scope Descriptors	Criteria					
**Aspects of WASH**[1]	Scope	Peer	Grey	Org	**Total**	%
	Water (only)	37	10	8	**55**	**41%**
	Sanitation (only)	15	10	4	**29**	**22%**
	Water and Sanitation	12	6	2	**20**	**15%**
	WASH	5	4	7	**16**	**12%**
	Water, Sanitation, Hygiene	3	2	1	**6**	**5%**
	Hygiene	1	1	0	**2**	**2%**
	Hygiene and Sanitation	2	2	0	**4**	**3%**
**Outcome Scope**[2]	Outcome	Peer	Grey	Org	**Total**	%
	Sustainability	48	23	17	**88**	**66%**
	Access	13	3	0	**16**	**12%**
	Health	4	0	0	**4**	**3%**
	Behavior Change	1	2	0	**3**	**2%**
	Coordination	2	1	0	**3**	**2%**
	Functionality	1	0	0	**1**	**1%**
	Policy	0	0	1	**1**	**1%**
**Context**[1]	Context	Peer	Grey	Org	**Total**	%
	Urban and Rural	9	10	11	**30**	**23%**
	Urban (Only)	18	7	2	**27**	**21%**
	Urban (Any)	27	16	13	**56**	**43%**
	Rural (Only)	44	15	8	**67**	**52%**
	Rural (Any)	53	24	19	**96**	**74%**
	None	4	1	2	**7**	**5%**
	Application	Peer	Grey	Org	**Total**	%
	Local	46	19	14	**79**	**59%**
	Regional	21	10	11	**42**	**32%**
	National	8	8	11	**27**	**20%**
	City	10	9	1	**20**	**15%**
	City-only	9	5	1	**15**	**11%**
	Sector	2	2	7	**11**	**8%**
	None / Unspecified	1	0	0	**1**	**1%**

Unless specified otherwise, each table represents counts of studies and % of total count of studies, n=133

Notes: 1: a-priori, deductive criteria; 2: open or emergent criteria

Note: Many descriptors represent non-exclusive criteria (i.e. studies can be classified under multiple attributes, % do not always sum to 100%)

Table SI 4-3: Results Summary Table - Application

Application Descriptors	Criteria

Project Application[1]

Project Application	Peer	Grey	Org	**Total**	%
Evaluation	26	15	15	**56**	**42%**
Case Study	38	9	4	**51**	**38%**
Planning	16	13	11	**40**	**30%**
Implementation	4	7	4	**15**	**11%**
None	0	0	1	**1**	**1%**

Location[1]

Region	Peer	Grey	Org	**Total**	%
East Africa	23	10	13	**46**	**40%**
South Asia	21	10	6	**37**	**32%**
West Africa	12	5	11	**28**	**24%**
South America	9	6	4	**19**	**17%**
East Asia	8	6	2	**16**	**14%**
Southeast Asia	8	6	2	**16**	**14%**
Central America	8	6	2	**16**	**14%**
South Africa	4		2	**6**	**5%**
Caribbean	2	2	1	**5**	**4%**
Oceania	1	3		**4**	**3%**
Central Asia	1	3		**4**	**3%**
North America	1			**1**	**1%**
East Europe		1		**1**	**1%**
Middle Africa	1			**1**	**1%**

Country	Peer	Grey	Org	**Total**	%
India	8	8	4	**20**	**15%**
Ghana	5	3	10	**18**	**14%**
Uganda	7	4	6	**17**	**13%**
Tanzania	5	6	2	**13**	**10%**
Nepal	7	3	1	**11**	**8%**
Kenya	7	1	2	**10**	**8%**
Nicaragua	3	4	2	**9**	**7%**
Bangladesh	6	1	1	**8**	**6%**
Burkina Faso	3		5	**8**	**6%**
Ethiopia	4	2	2	**8**	**6%**
Indonesia	3	4	1	**8**	**6%**
Bolivia	4	2	1	**7**	**5%**
Peru	1	4	2	**7**	**5%**
Honduras	1	3	2	**6**	**5%**
Malawi	2	1	3	**6**	**5%**
Mexico	3	3		**6**	**5%**

	Philippines	2	3	1	**6**	**5%**
	South Africa	4		2	**6**	**5%**
	Vietnam	1	5		**6**	**5%**
	Brazil	2	2	1	**5**	**4%**
	Colombia	3	1		**4**	**3%**
	Dominican Republic	1	2	1	**4**	**3%**
	Mozambique		1	3	**4**	**3%**
	Rwanda	2		2	**4**	**3%**
	Senegal		2	2	**4**	**3%**
	Costa Rica	1	2		**3**	**2%**
	Lao	2	1		**3**	**2%**
	Niger	1	1	1	**3**	**2%**
	Panama		3		**3**	**2%**
	Timor-Leste	2	1		**3**	**2%**
	Benin		1	1	**2**	**2%**
	Cambodia	2			**2**	**2%**
	China		2		**2**	**2%**
	Columbia	1		1	**2**	**2%**
	Fiji	2			**2**	**2%**
	Guatemala	1		1	**2**	**2%**
	Haiti	1	1		**2**	**2%**
	Madagascar		1	1	**2**	**2%**
	Nigeria	1	1		**2**	**2%**
	Pakistan	2			**2**	**2%**
	Zimbabwe	2			**2**	**2%**
	Argentina	1			**1**	**1%**
	Canada	1			**1**	**1%**
	Egypt			1	**1**	**1%**
	Jordan			1	**1**	**1%**
	Kyrgyz Republic		1		**1**	**1%**
	Kyrgyzstan		1		**1**	**1%**
	Laos	1			**1**	**1%**
	Liberia			1	**1**	**1%**
	Mali	1			**1**	**1%**
	Moldova		1		**1**	**1%**
	Morocco		1		**1**	**1%**
	Palestine			1	**1**	**1%**
	Papua New Guinea		1		**1**	**1%**
	Paraguay		1		**1**	**1%**
	Sudan			1	**1**	**1%**
	Sweden		1		**1**	**1%**
	Tajikistan				**1**	**1%**
	Thailand	1			**1**	**1%**
	Zambia			1	**1**	**1%**
**Reporting of Impacts[2]**	Study Impacts	Peer	Grey	Org	**Total**	%
	Uptake	7	5	4	**16**	**12%**
	Services	4	3	2	**9**	**7%**
	Policy	2	2	4	**8**	**6%**

	Coordination	2		3	**5**	**4%**
	Behavior Change	2	2		**4**	**3%**
	Financial		1	1	**2**	**2%**
	Users	1		1	**2**	**2%**
	Health	1			**1**	**1%**
	Access	1			**1**	**1%**

Unless specified otherwise, each table represents counts of studies and % of total count of studies, n=133

Notes: 1: a-priori, deductive criteria; 2: open or emergent criteria

Note: Many descriptors represent non-exclusive criteria (i.e. studies can be classified under multiple attributes, % do not always sum to 100%)

© 2019 by the authors. Submitted for possible open access publication under the terms and conditions of the Creative Commons Attribution (CC BY) license (http://creativecommons.org/licenses/by/4.0/).
